# Supplementary material for: To develop online platform and determine its effectiveness in ENHANCING DIABetes knowledge among diabetes patients in primary CARE clinic (Enhancing-Diab-Care Study): Study protocol
Source: PLoS One. 2025 May 5;20(5):e0323102. doi: 10.1371/journal.pone.0323102 (PMC12052173; doi:10.1371/journal.pone.0323102)
Supplement: S1 — (ZIP) [file pone.0323102.s001.zip › Enhancing-Diab-Care Study Questionnaire .docx]

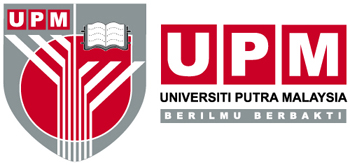

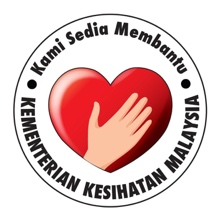


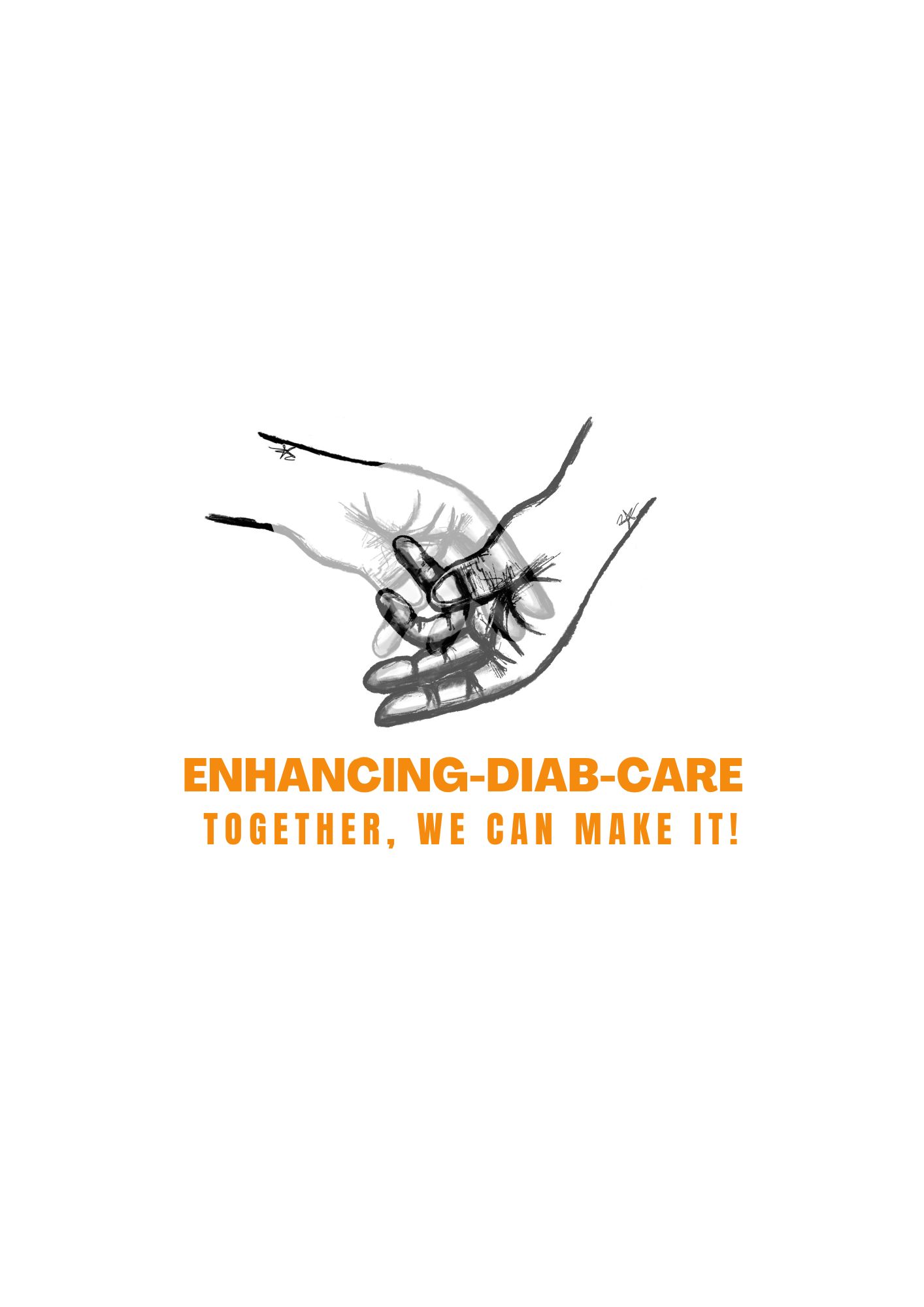


To develop online education video platform and determine its effectiveness in **ENHANCING DIAB**etes knowledge among type 2 diabetes patients in primary **CARE** clinics

(Enhancing-Diab-Care Study)

*Pembangunan platform video pendidikan dalam talian dan keberkesanannya dalam meningkatkan pengetahuan kencing manis di kalangan pesakit kencing manis jenis 2 di klinik penjagaan primer (Enhancing-Diab-Care Study)*

**SPECIFICATION QUESTIONS**

Please fill in an empty space or mark ✓ on the respective compartment

All information provided is confidential and will not be disclosed to anyone unrelated.

The information provided will be used for research purposes only.

| DATE OF RECRUITMENT (DD/MM/YYYY): |  |  | **/** |  |  | **/** | **2** | **0** |  |  |
| --- | --- | --- | --- | --- | --- | --- | --- | --- | --- | --- |
| PATIENT ID: |  |  |  |  |  |  |  |  |  |  |

| **Part 1: Social Demographic Background** | | |
| --- | --- | --- |
|  | Birth Date | ______day ______month ____year |
|  | Gender |  Male   Female |
|  | Ethnic |  Malay   Chinese   Indian   Others |
|  | Educational status |  Never went to school   Primary education   Secondary education   Diploma/ University |
|  | Marriage Status |  Single   Married   Windowed |
|  | Are you working? |  Yes   No |
|  | Household income | Please state, RM _________ per month |

| **Part 2: Medical Information** | | |
| --- | --- | --- |
|  | Were you diagnosed with high blood pressure? |  Yes   No   Unsure |
|  | Were you diagnosed with high cholesterol? |  Yes   No   Unsure |
|  | Were you diagnosed with obsesity? |  Yes   No   Unsure |
|  | Had you smoked? |  Yes   Never   Yes. But stopped for less than 6 months   Yes. But stopped for more than 6 months |
|  | Which year and month were you diagnosed with diabetes? | Please state, ______year ________month |
|  | Do you have any admission history due to complication of diabetes? |  Yes   No   Unsure |
|  | Have you ever met with a diabetic health educator? |  Yes  If Yes, when was the last time you met a diabetic health educator?  (month) _________(year) ________  If Yes, please select the advice/education you have received:   Risk of diabetes complications  Specify date: ____________________   Self-care for diabetes  Specify date: ____________________   Testing glucose/sugar levels  Specify date: ____________________   Drug intake  Specify date: ____________________   Dietary nutrition  Specify date: ____________________   Activity and exercise  Specify date: ____________________   Foot care  Specify date: ____________________   Stress / emotional management  Specify date: ____________________   Insulin injection  Specify date: ____________________   No |
|  | Do you have a glucometer?  (Note: Glucometers are self-testing devices to measure your blood glucose/sugar levels) |  Yes   No |
|  | Do you check your glucose/sugar levels with a glucometer at home regularly? |  Yes  If yes, please mark and indicate how often?  _________ in a week  _________ in a month   No |
|  | Do you have a sphygmomanometer (blood pressure monitoring machine) at home? |  Yes   No |
|  | Do you know how to check blood pressure at home? |  Yes   No |
|  | Specify the frequency you check your blood pressure at home in a week? | Please specify, __________in a week |
|  | Are you monitoring your weight? |  Yes  If Yes, how often? ________ times a week/month   No |
|  | Are you monitoring your waist circumference? |  Yes  If Yes, how often? ________ times a week/month   No |
|  | Do you practice healthy “quarter-quarter-half” plates during daily feeding?  Note: A healthy plate “quarter-quarter-half” is a plate divided into 3 parts:   1. A quarter of plate with a source of carbohydrates, 2. A quarter of plates with sources of protein and fat, 3. Half a plate with vegetables and fruits |  Yes   No   Once in a while. |
|  | Do you know how to take care for your feet? |  Yes  If Yes, how often? ________ times a week/month   No |
|  | Do you know you need to check your eyes once a year or more frequently depending on your eye condition to avoid eye complications? |  Yes   No |
|  | Have you ever met with an ophthalmologist for your eye examination? |  Ya  If Yes, specify the latest follow up date ________________   No |
|  | Do you see a dentist for dental examination every year? |  Yes   No |

| **Bahagian 3: DKT Malay Version**  (Sila tandakan √ pada petak yang berkenaan) | | | | |
| --- | --- | --- | --- | --- |
| **No.** | **Statement** | **True** | **False** | **Not sure** |
| 1. | Pemakanan diabetes adalah diet yang sihat untuk kebanyakan orang. |  |  |  |
| 2. | Glikosilat hemoglobin ( HbA1c) adalah ujian untuk mengukur purata kadar gula dalam darah sepanjang tempoh seminggu yang lepas. |  |  |  |
| 3. | Semangkuk nasi putih mempunyai kandungan karbohidrat yang lebih tinggi berbanding sekeping roti putih. |  |  |  |
| 4. | Jus oren mempunyai lebih banyak kandungan lemak berbanding susu rendah lemak. |  |  |  |
| 5. | Pemeriksaan air kencing dan pemeriksaan darah adalah sama baik untuk mengukur kadar gula dalam darah. |  |  |  |
| 6. | Jus buah-buahan tanpa gula boleh meningkatkan kadar gula dalam darah. |  |  |  |
| 7. | Satu tin minuman bergas kurang gula boleh digunakan untuk merawat kadar gula rendah di dalam darah. |  |  |  |
| 8. | Menggunakan minyak zaitun dalam masakan boleh mengurangkan kadar kolesterol dalam darah. |  |  |  |
| 9. | Senaman secara kerap boleh membantu mengurangkan tekanan darah yang tinggi. |  |  |  |
| 10. | Bagi pesakit yang baik kawalan gulanya, senaman tidak memberi kesan terhadap kadar gula dalam darah. |  |  |  |
| 11. | Jangkitan kuman berkemungkinan menyebabkan peningkatan kadar gula di dalam darah. |  |  |  |
| 12. | Memakai kasut yg bersaiz besar dari kebiasaan boleh mengelakkan ulser kaki. |  |  |  |
| 13. | Makanan rendah lemak mengurangkan risiko masalah jantung. |  |  |  |
| 14. | Rasa kebas dan sesemut merupakan gejala penyakit saraf. |  |  |  |
| 15. | Masalah paru-paru kebiasaannya dikaitkan dengan penyakit kencing manis |  |  |  |
| 16. | Sekiranya anda sakit selesema, anda patut memeriksa kadar gula dengan lebih kerap. |  |  |  |
| 17. | Pemeriksaan kesihatan secara berkala boleh membantu untuk mengesan tanda-tanda awal komplikasi kencing manis. |  |  |  |
| 18. | Menghadiri temu janji kencing manis anda akan mengelakkan anda dari mendapat komplikasi kencing manis. |  |  |  |
| **Soalan 19 dan 20 hanya untuk pesakit yang mengambil insulin** | | | | |
| 19. | Kadar gula yang tinggi di dalam darah mungkin disebabkan oleh pengambilan insulin yang terlalu banyak. |  |  |  |
| 20. | Jika anda mengambil insulin pada waktu pagi dan tidak bersarapan, kadar gula di dalam darah anda akan berkurangan (hipoglicemia) |  |  |  |

| **Bahagian 4: DES-28**  (Sila tandakan √ pada petak yang berkenaan) | | | | | | |
| --- | --- | --- | --- | --- | --- | --- |
| **No.** | **Kenyataan** | **Sangat setuju** | **Setuju** | **Neutral** | **Tidak setuju** | **Sangat tidak setuju** |
|  | Saya tahu bahagian apa dalam penjagaan diabetes yang saya berpuas hati. |  |  |  |  |  |
|  | Saya tahu bahagian apa dalam penjagaan diabetes yang saya tidak berpuas hati. |  |  |  |  |  |
| **No.** | **Kenyataan** | **Sangat setuju** | **Setuju** | **Neutral** | **Tidak setuju** | **Sangat tidak setuju** |
|  | Saya tahu bahagian apa dalam penjagaan diabetes yang saya bersedia untuk tukar. |  |  |  |  |  |
|  | Saya tahu bahagian apa dalam penjagaan diabetes yang saya tidak bersedia untuk tukar. |  |  |  |  |  |
|  | Saya boleh memilih matlamat diabetes yang realistik. |  |  |  |  |  |
|  | Saya tahu yang mana satu matlamat diabetes yang sangat penting bagi saya |  |  |  |  |  |
|  | Saya tahu perkara tentang diri saya sama ada menolong atau menghalang saya daripada mencapai matlamat diabetes saya |  |  |  |  |  |
|  | Saya boleh mengeluarkan idea yang bagus untuk menolong saya mencapai matlamat saya. |  |  |  |  |  |
|  | Saya mampu menjadikan matlamat diabetes kepada sesuatu rancangan yang boleh dijalankan. |  |  |  |  |  |
|  | Saya mampu mencapai matlamat diabetes saya apabila saya buka fikiran saya. |  |  |  |  |  |
|  | Saya tahu halangan yang menyukarkan saya untuk mencapai matlamat diabetes. |  |  |  |  |  |
|  | Saya boleh berfikir pelbagai cara untuk mengatasi halangan tersebut. |  |  |  |  |  |
|  | Saya boleh mencuba pelbagai cara untuk mengatasi halangan tersebut. |  |  |  |  |  |
|  | Saya mampu memilih jalan yang terbaik bagi mengatasi halangan tersebut. |  |  |  |  |  |
|  | Saya boleh memberitahu bagaimana perasaan saya apabila menhidapi diabetes. |  |  |  |  |  |
|  | Saya boleh memberitahu perasaan saya dalam menjaga diabetes. |  |  |  |  |  |
|  | Saya tahu bahawa diabetes boleh menyebabkan saya berasa tertekan dalam kehidupan saya. |  |  |  |  |  |
|  | Saya tahu cara positif untuk menghadapi tekanan yang disebabkan oleh diabetes. |  |  |  |  |  |
|  | Saya tahu cara negatif untuk menghadapi tekanan yang disebabkan oleh diabetes. |  |  |  |  |  |
|  | Saya mampu mengatasi tekanan yang disebabkan oleh diabetes. |  |  |  |  |  |
|  | Saya tahu dimana saya boleh mendapatkan sokongan bagi menghadapi diabetes. |  |  |  |  |  |
|  | Saya boleh meminta sokongan apabila saya memerlukan bagi menghadapi diabetes. |  |  |  |  |  |
|  | Saya boleh mendorong diri saya dalam menghadapi diabetes. |  |  |  |  |  |
|  | Saya tahu perkara yang membuatkan saya sentiasa bermotivasi dalam mengawal diabetes. |  |  |  |  |  |
| **No.** | **Kenyataan** | **Sangat setuju** | **Setuju** | **Neutral** | **Tidak setuju** | **Sangat tidak setuju** |
|  | Saya boleh memotivasikan diri saya untuk mengawal diabetes. |  |  |  |  |  |
|  | Saya cukup tahu tentang diabetes supaya saya dapat membuat pilihan yang betul dalam menjaga diri saya |  |  |  |  |  |
|  | Saya cukup tahu tentang diri saya kerana saya yang akan membuat pilihan yang betul dalam penjagaan diabetes. |  |  |  |  |  |
|  | Saya berpendapat bahawa adalah berbaloi bagi saya mengunakan masa saya untuk menukar cara menangani diabetes |  |  |  |  |  |

| **Bahagian 5:** | | | | | |
| --- | --- | --- | --- | --- | --- |
| 1. How many times have you watched this video for the last 3 months? | | | | | Please specify _________ times |
| Do you think this video is beneficial?  (Sila bulatkan) | **Extremely agree** | **Agree** | **Neutral** | **Disagree** | **Extremely disagree** |

| **Bahagian 6: Maklumat Klinikal (Untuk diisi oleh Pembantu Penyelidik)** | | |
| --- | --- | --- |
|  | Height | _________ meter |
|  | Weight | _________ kg |
|  | BMI | _________ kg/m^2^ |
|  | Waist circumference | _________ cm |
|  | Latest HbA1c | _________ % (Tarikh: _____________ ) |
|  | Latest LDL level | _________ mmol/L (Tarikh: ___________ ) |
|  | Latest HDL level | _________ mmol/L (Tarikh: ___________ ) |
|  | Latest Triglyceride level | _________ mmol/L (Tarikh: ___________ ) |
|  | Blood pressure | _________ mmHg |
