# Supplementary material for: To develop online platform and determine its effectiveness in ENHANCING DIABetes knowledge among diabetes patients in primary CARE clinic (Enhancing-Diab-Care Study): Study protocol
Source: PLoS One. 2025 May 5;20(5):e0323102. doi: 10.1371/journal.pone.0323102 (PMC12052173; doi:10.1371/journal.pone.0323102)
Supplement: S1 — (ZIP) [file pone.0323102.s001.zip › PIS BI .docx]

**PATIENT INFORMATION SHEET AND INFORMED CONSENT FORM**

*(For adult subjects and interventional studies)*

1. **Title of study**: Development of online education video platform and determine its effectiveness in **ENHANCING DIAB**etes knowledge among type 2 diabetes patients in primary **CARE** clinics **(Enhancing-Diab-Care Study)**
2. **Name of investigator and institution:**

1. Dr Thew Hui Zhu, Department of Family Medicine, University Putra Malaysia
2. Associate Prof Dr. Cheong Ai Theng, Department of Family Medicine, University Putra Malaysia
3. Prof Dr. Sazlina Shariff Ghazali, Department of Family Medicine, University Putra Malaysia
4. Dr. Lim Poh Ying, Department of Community Health, Universiti Putra Malaysia
5. Dr Wong Pin Foo, Klinik Kesihatan Cheras Baru
6. Associate Prof Dr. Aneesa Abdul Rashid, Department of Family Medicine, University Putra Malaysia
7. **Name of sponsor:** Grant Universiti Putra Malaysia – GP-IPM/2022/9714000
8. **Introduction:**

You are invited to participate in a research study starting September 2023 because you are 18 years and above, have been diagnosed and followed up for type 2 diabetes for at least six months, and your HbA1c is greater than 8%. Only one of you or your family members can be in this study if they are also diagnosed and followed up for type 2 diabetes. You need to be able to understand Malay and have internet access at home.The details of the research study are described in this document. It is important that you understand why the research is being done and what it will involve. Please take your time to read through and consider this information carefully before you decide if you are willing to participate. Ask the study staff if anything is unclear or if you’d like more information. After you are properly satisfied that you understand this study, and that you wish to participate, you must sign this informed consent form. To participate in this study, you may be required to provide your demographic information to the invigilator to reach you regarding your progress and appointment reminders.

Your participation in this study is voluntary. You do not have to be in this study if you do not want to. If you are undecisive, you will be given one week to consider for the participation of the study. You may also refuse to answer any questions you do not want to answer. If you volunteer to be in this study, you may withdraw from it at any time. If you withdraw, any data collected from you up to your withdrawal will still be used for the study. Your refusal to participate or withdrawal will not affect any medical or health benefits to which you are otherwise entitled.

This study has been approved by the Medical Research and Ethics Committee, Ministry of

Health Malaysia.

1. **What is the purpose of the study?**

The purpose of this study is to develop web-based education videos for type 2 diabetes patients in primary care and investigate its effectiveness in enhancing diabetes knowledge among type 2 diabetes patients. This research is necessary because it can enhance diabetes knowledge and self-empowerment among diabetes patients.

Previously you had to see a diabetes educator with a few appointments with her to understand what diabetes is and how to take care of your diabetes. In this study, you will be given a set of online diabetes educational videos to enhance your knowledge whenever is convenient for you.

A total of 232 subjects like you from Klinik Kesihatan Cheras Baru, Pejabat Kesihatan Cheras, Jabatan Kesihatan Wilayah Persekutuan Kuala Lumpur dan Putrajaya will be participating in this study. The whole study will last about 6 months and your participant will be at the enrollment, first month, third month and at sixth months.

1. **What will happen if I decide to take part?**

If you agree to take part in this study, you will be invited for a session to answer a questionnaire and health assessment. The researcher will ask you to sign a consent form, followed by filling in a questionnaire about your background, medical history and on diabetes knowledge and empowerment. Information on health assessments involving weight, height, body mass index, waist circumference, blood pressure and blood tests on HbA1c and fasting lipid profile will be taken on your latest medical record. This may take about 20 minutes.

This is a 6-month study that has two groups. If you agree to participate, you will be randomly allocated to one of the groups. Group 1 will use a series of online educational videos (12 videos of about 5 to 10 minutes duration for each), in addition to the usual care. These videos are as follows:

1) An introduction to Enhancing Diabetes Care

2) A guide to screening, diagnosis and HbA1c test

3) Oral antidiabetic medications
4) Injection form antidiabetic medications and injection technique
5) Self-monitoring blood glucose (SMBG)
6) Hypoglycemia

7) Dietary
8) Self-care
9) Exercise
10) Understanding complications and prevention

11) Recap and roadmap to a better future.
12) Real-life journey: a type 2 diabetes patient shares his testimony

The research assistants who have already been trained by the researcher, he will teach you how to assess and use the web-based online education videos. You will need to watch all the videos. In between the following 4 weeks, research assistant will remind you and follow up your progress and assist you if any technical problems with the web-based education videos via phone call or message. Group 2 is a comparison group who will receive usual diabetes care.

In the third month of the study and the sixth month of the study, you will need to return to the clinic for follow-ups. The research assistants will distribute diabetes knowledge questionnaire again to you to assess your knowledge. This may take about 10 minutes. The latest blood test results of HbA1c and fasting lipid profile will be taken from your latest medical record on the sixth month of the study. This is a basic diabetes education video for self-care and education, and no further treatment will be given after the study has been completed. You will continue your diabetes follow up as usual.

1. **What are my responsibilities when taking part in this study?**

It is important that you answer all the self-administrated questionnaires honestly and completely.

1. **What are the potential risks and side effects of being in this study?**

During the study, sometimes, you may be taken more time to answer the questionnaires.

1. **What are the benefits of being in this study?**

This research may or may not directly benefit you. Information gained from this research will help in increasing your knowledge of diabetes and possibly improve your diabetes self-care. There is potential for online educational videos to be implemented systematically in all primary care clinics to facilitate patient self-management and reduce the risk of diabetic complications.

1. **Who is funding the research?**

This study is funded by Grant Universiti Putra Malaysia (Ref.:GP-IPM/2022/9714000). The Sponsor has overall responsibility for the running of the study and will financially compensate the time spent by the study staff, use of facilities, etc., for including you in the study. You will be given a token of appreciation upon completion of the study.

1. **Can the research or my participation be terminated early?**

Yes. Your participation in this study is voluntary. You do not have to be in this study if you do not want to. You may also refuse to answer any questions you do not want to answer. If you volunteer to be in this study, you may withdraw from it at any time. If you withdraw, any data collected from you up to your withdrawal will still be used for the study. Your care at the clinic will not be affected.

1. **Will my medical information be kept private?**

You (the participants) will not be identified through personal identifiable information but will be given a study identification number such as 1, 2, 3 etc. All investigators and study site team involved with this study must comply with the requirements of the appropriate data protection legislation with regard to the collection, storage, processing and disclosure of personal information. All the information we will collect in paper copies will be stored under lock and key in Universiti Putra Malaysia, while the electronic data will be anonymized and can only be accessed with a secure password in laptops for this research kept in Universiti Putra Malaysia. Access to unidentifiable participant data will be restricted to individuals from the research team treating the participants, representatives of the sponsor(s) and representatives of regulatory authorities. When publishing or presenting the study results, your identity will not be revealed. If you withdraw from the study, we will keep the unidentified information about you that we have already obtained. Data will be stored for 5 years and will be destroyed after the period of storage.

1. **Would I be informed about the findings of this study?**

If you wish to be informed about the study findings, please inform the investigators. The investigators would be happy to share the findings with you.

1. **What happens when new important information about this study becomes available?**

If new important information about this study becomes available, you will be notified and consent will be re-taken.

1. **Who should I call if I have questions?**

If you have any questions about the study, please contact the investigators:

- Dr Thew Hui Zhu (012-405771)
- Associate Prof Dr Cheong Ai Theng (0124562389)
- Prof Dr Sazlina Shariff Ghazali (0122325659)

If you have any questions about your rights as a participant in this study, please contact: The Secretary, Medical Research & Ethics Committee, Ministry of Health Malaysia, at telephone number 03-3362 8407 / 8205 / 8888.

**INFORMED CONSENT FORM**

Title of Study: Development of online education video platform and determine its effectiveness in **ENHANCING DIAB**etes knowledge among type 2 diabetes patients in primary **CARE** clinics (Enhancing-Diab-Care Study)

By signing below, I confirm the following:


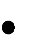
 I have been given oral and written information for the above study and have read and understood the information given.


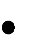
 I have had sufficient time to consider participation in the study and have had the opportunity to ask questions and all my questions have been answered satisfactorily.


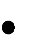
 I understand that my participation is voluntary, and I can at any time free withdraw from the study without giving a reason and this will in no way affect my future treatment. I am not taking part in any other research study at this time. I understand the risks and benefits, and I freely give my informed consent to participate under the conditions stated. I understand that I must follow the study doctor’s (investigator’s) instructions related to my participation in the study.


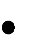
 I understand that study staff, qualified monitors and auditors, the sponsor, or its affiliates, and governmental or regulatory authorities, have direct access to my medical record to make sure that the study is conducted correctly, and the data are recorded correctly. All personal details will be treated as STRICTLY CONFIDENTIAL


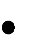
 I will receive a copy of this subject information/informed consent form signed and dated to bring home.

**Subject:**

| Signature: | I/C number: |
| --- | --- |
| Name: | Date: |

**Investigator conducting informed consent:**

| Signature: | I/C number: |
| --- | --- |
| Name: | Date: |

**Impartial witness:** *(Required if subject is illiterate and contents of patient information sheet is orally communicated to subject)*

| Signature: | I/C number: |
| --- | --- |
| Name: | Date: |
