# Supplementary material for: To develop online platform and determine its effectiveness in ENHANCING DIABetes knowledge among diabetes patients in primary CARE clinic (Enhancing-Diab-Care Study): Study protocol
Source: PLoS One. 2025 May 5;20(5):e0323102. doi: 10.1371/journal.pone.0323102 (PMC12052173; doi:10.1371/journal.pone.0323102)
Supplement: S1 — (ZIP) [file pone.0323102.s001.zip › Study Protocol Version 4.0.docx]

# Study Protocol

To develop online platform and determine its effectiveness in ENHANCING DIABetes knowledge among diabetes patients in primary CARE clinic (Enhancing-Diab-Care Study)

**Protocol number: 22-02453-OKN**

**Name and Institution of Principal investigator:**

Dr Thew Hui Zhu, Department of Family Medicine, University Putra Malaysia

**Name and Institution of Co-Investigators:**

1. Associate Prof Dr. Cheong Ai Theng, Department of Family Medicine, Universiti Putra Malaysia
2. Prof Dr. Sazlina Shariff Ghazali, Department of Family Medicine, Universiti Putra Malaysia
3. Dr. Lim Poh Ying, Department of Community Health, Universiti Putra Malaysia
4. Dr Wong Pin Foo, Klinik Kesihatan Cheras Baru
5. Associate Prof Dr. Aneesa Abdul Rashid, Department of Community Health, Universiti Putra Malaysia

**Name and address of Sponsor:**

Grant Universiti Putra Malaysia – GP-IPM/2022/9714000

**Study site/s:**  Klinik Kesihatan Cheras Baru

Table of Contents

[Study Protocol 1](#_Toc119939045)

[Background and Significance 3](#_Toc119939046)

[1.1 Background 3](#_Toc119939047)

[1.2 Research Question 4](#_Toc119939048)

[1.3 Conceptual Framework 4](#_Toc119939049)

[2. Objective 5](#_Toc119939050)

[2.1 General objective 5](#_Toc119939051)

[2.2 Specific objective 5](#_Toc119939052)

[3. Methodology 6](#_Toc119939053)

[3.1 Study Type and Design 6](#_Toc119939054)

[3.2 Study Population 6](#_Toc119939055)

[3.3 Inclusion Criteria 6](#_Toc119939056)

[3.4 Exclusion Criteria 7](#_Toc119939057)

[3.5 Withdrawal Criteria 7](#_Toc119939058)

[3.6 Sample Size 7](#_Toc119939059)

[3.7 Recruitment and randomisation 7](#_Toc119939060)

[3.8 Study Duration and Timeline 8](#_Toc119939061)

[3.9 Data collection method 8](#_Toc119939062)

[3.10 Study Instrument 10](#_Toc119939063)

[3.11 Operational Definition 12](#_Toc119939064)

[3.12 Statistical Analysis Plan 13](#_Toc119939065)

[3.13 Risk and benefit to study participants 14](#_Toc119939066)

[3.14 Ethics of Study 14](#_Toc119939067)

[3.15 Data storage, privacy and confidentiality 15](#_Toc119939068)

[3.16 Informed Consent/Assent Process 15](#_Toc119939069)

[3.17 Conflict of Interest 15](#_Toc119939070)

[3.18 Publication Policy 16](#_Toc119939071)

[3.19 Termination of Study 16](#_Toc119939072)

[3.20 Study Outcome 16](#_Toc119939073)

[The primary outcome of interest are diabetes knowledge and diabetes empowerment. 16](#_Toc119939074)

[The secondary outcome is clinical outcome (HbA1c, blood pressure, fasting lipid profile, BMI, waist circumference) among intervention and control groups at baseline. 16](#_Toc119939075)

[References 17](#_Toc119939076)

# Background and Significance

## 1.1 Background

Diabetes is a growing global health concern, with its highest prevalence found in low- and middle-income countries and accounts for 1.5 million deaths per year. In Southeast Asia, type 2 diabetes is now the most prevalent disease. (1) According to the International Diabetes Federation (IDF), 34 million people in south-east Asia have diabetes. According to estimates, the number will rise to over 55 million by 2035. (1) In Malaysia, there are approximately 3.9 million people with diabetes, according to the National Health and Morbidity Survey 2019. This rate has increased from 13.4% in 2015 to 18.3% in 2019.

Alternatively, that is approximately one-fifth of the adult population in Malaysia. (2) Moreover, there is a high prevalence of poor glycaemic control with 7.9% mean HbA1c levels. (2) Several studies show that Malaysians with poor glucose control engage in poor self-care practices and have a limited understanding of diabetes. (3-6) Additionally, there are also insufficient diabetes educators to deal with a large number of diabetes patients. (7) Furthermore, COVID-19 has had a significant impact on the entire health care system in recent years. Primary care and hospital practice are currently overburdened with diagnostic testing, monitoring, and management of COVID-19 care, to the point that our routine maintenance for non-communicable diseases can be compromised. (8) Given these factors, there is an urgent need for practical solutions.

Studies have demonstrated that diabetes education improves self-management and glucose control among people with low health literacy. At the same time, clinicians have less time and resources to disseminate information. (9, 10) The number of diabetic education classes is disappointingly low, particularly among the patients with lower socioeconomic classes, those who have not yet developed diabetes complications, and during the COVID19 pandemic when most patients are unwilling to stay in the hospital for longer.

Diabetes self-management programs include health education, a critical component for the management and treatment of chronic diseases. (11) This is the principle that underpins the theory of knowledge, awareness, and practices (KAP) of patients with diabetes are considered the most important factors for assessing health education outcomes. As outlined by the KAP theory, the process of human behaviour change can be divided into three steps: acquiring knowledge, creating attitudes and beliefs, and changing behaviours, during which human health behaviours can also be transformed. (11,12) The use of knowledge can assist patients in making informed decisions about their health when dealing with non-communicable diseases such as diabetes. By empowering them, patients can increase their self-efficacy and improve their self-confidence. Utilising the media in publicity campaigns, creating and creating educational materials, and educating people with diabetes and their families should be part of this effort. (13) Diabetes education and awareness programs will benefit both healthy subjects as prevention measures and help people with diabetes better control their condition. (14,15) It is essential to provide specific and practical guidance for maintaining NCDs’ critical health and community services. National guidance is required to develop and use digital health solutions in NCD care, selfcare, at-home care, and peer support. Yet, with the challenges of the COVID-19 pandemic facing us in the 21st century, it is vital to evaluate how medical information is delivered effectively through health promotion in the practice of new norms. The systematic reviews on using technology to facilitate diabetes self-management reported a significant reduction in glycated haemoglobin and cost-effectiveness. With Internet-delivered diabetes education, many individuals have easier access to the material, and they can take their time with the learning process. (16-18)

## 1.2 Research Question

1. How effective are diabetes education videos in enhancing diabetes knowledge among type 2 diabetes patients?
2. Can diabetes education videos improve clinical outcome among type 2 diabetes patients?

## 1.3 Conceptual Framework

*Figure 1 conceptual framework*


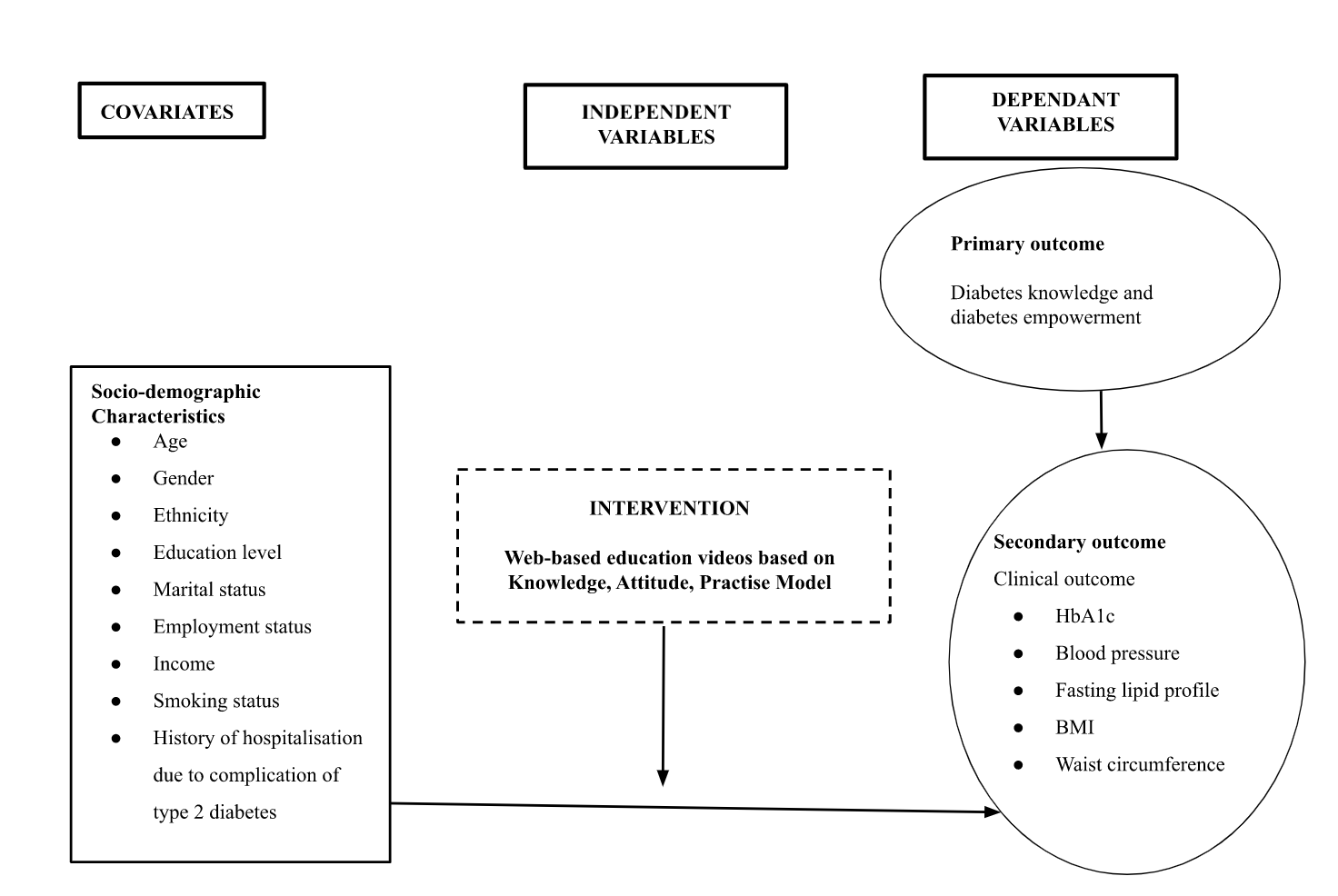


# Objective

## General objective

To investigate the effectiveness of web-based education videos that include tailored information on knowledge to educate type 2 diabetes patients in primary care.

## Specific objective

1. To develop web-based education videos for type 2 diabetes patients in primary care.
2. To identify the sociodemographic characteristics (age, gender, ethnicity, education level, marital status, employment status, income, smoking status, history of hospitalisation due to complication of type 2 diabetes) of the type 2 diabetes patient in the primary care clinic among intervention and control groups at baseline.
3. To determine the knowledge scores and clinical outcome (HbA1c, blood pressure, fasting lipid profile, BMI, waist circumference) among intervention and control groups at baseline.
4. To investigate the effectiveness of web-based education videos and compare on the knowledge, diabetes empowerment and clinical outcome (HbA1c, blood pressure, fasting lipid profile, BMI, waist circumference) at baseline, 3months and 6 months between and within the intervention and control groups.

# Methodology

## Study Type and Design

The study design that will be used in this study is a Blocked Randomized Control Trial (RCT) ratio one to one. The samples will be from Klinik Kesihatan Cheras Baru. The study will be conducted and evaluated by the requirement in the Consolidated Standards of Reporting Trials (CONSORT) Statement. (19)

## Study Population

The study population consists of type 2 diabetes patients through walk-in and follow-up clinic registrations in the primary care clinics from 1^st^ June 2024 to 31th December 2024.

## Inclusion Criteria

Those eligible for enrolling are type 2 diabetes patients aged 18 years or older, who have been diagnosed and followed up for type 2 diabetes for at least six months, have an HbA1c greater than 8% at the time of enrolment, and only one family member from each household will participate in the study. Furthermore, they should be able to understand Malay and have internet access at home.

## Exclusion Criteria

Exclusion criteria include patients who are acutely ill, have psychiatric illness, who are bedridden and rely on nursing care to perform daily activities and who are intellectually disabled, as determined by a community-centred board.

## Withdrawal Criteria

Patients whom who wish to withdraw from the study can withdraw from the study anytime by informing principal investigator.

## Sample Size

Sample size was calculated based on two mean groups formula (20) , mean±sd of knowledge differences for intervention group and control group were 0.7±2.21 and -0.30±2.33 (16), 80% of power, 5% of risk error, 30% attrition rate, the sample size needed per group is 116 (total is 232).

## Recruitment and randomisation

The study design that will be used in this study is a Blocked Randomized Control Trial (RCT) ratio one to one.

## Study Duration and Timeline

The duration of data collection of the research is 6 months, from 1^st^ June 2024 to 31^th^ December 2024.

## Data collection method

Data will be collected by the research by selecting type 2 diabetes patients from the baseline. These type 2 diabetes patients will have to meet the inclusion and exclusion criteria, once it is fulfilled the type 2 diabetes patients will be recruited. A research assistant will approach patients on the day of their follow-up visit at Klinik Kesihatan Cheras Baru. As part of the consent process, the investigator will explain and provide the Patient Information Sheet to the participant and an agreement to receive calls and messages from the researcher for research purposes. They will keep their phone numbers private and will only be contacted or messaged every four weeks to remind them to complete the videos watching and follow-up appointment. The patients will, however, be given one week to consider their participation if they are still deciding on the recruitment day. We will conduct a one-to-one ratio randomization control trial with the control and intervention groups.

During the same day, all participants will be given a hard copy self-administered questionnaire by research assistance to complete and their latest clinical profile (height, weight, BMI, waist circumference, blood pressure, latest blood test for HbA1c and fasting lipid profiles) will be taken from their medical records. The self-administered questionnaire will take about 20minutes to complete. This stage will be considered as baseline assessment for both control and intervention groups. After the questionnaire, both groups will continue their follow up in the clinic like usual. The intervention group will take place after all the participants complete the questionnaire. The participants will be told about the structure of the program. There will be a research assistant who the researcher has already trained to assist and teach the participants how to assess and use the web-based online education videos. They will need to watch all the videos. The research assistant will follow up with their progress and remind them to complete the video in every four weeks via phone call.

After three months of the intervention on the first week of September (2/9/2024 to 6/9/2024), both of the intervention and control group participants will return to the clinics for follow up. During the follow-up, participants will be given a self-administered questionnaire to answer.

At six months’ time point from the baseline on the last week of December (23/12/2024 to 31/12/2024), both of the intervention and control group participants will return to the clinics for follow up. During the follow-up, participants will be given a self-administered questionnaire to answer and their latest clinical profile (height, weight, BMI, waist circumference, blood pressure, latest blood test for HbA1c and fasting lipid profiles) will be taken from their medical records. This is a basic diabetes education video for self-care and education, and no further treatment will be given after the study has been completed. You will continue your diabetes follow up as usual.

*Figure 2: Flow of the process*


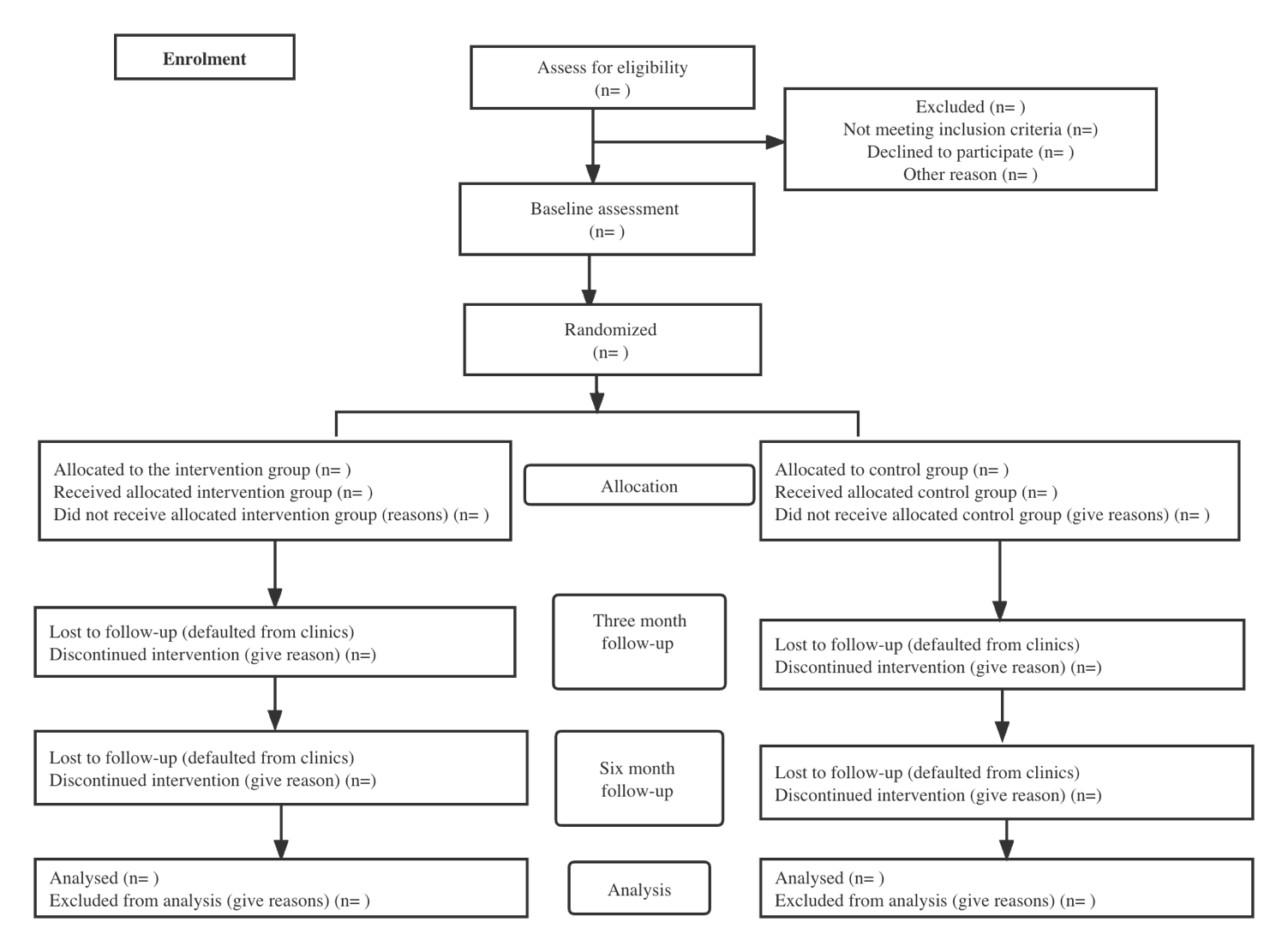


Collection of questionnaires port write up and dissemination of result

## Study Instrument

Self-administered questionnaire which will be distributed to respondent in Malay language upon initiation of recruitment as the baseline. This questionnaire will be divided into following sections.

**Section A for sociodemographic factors**

Section A will elicit on socio-demographic factors, such as age, gender, ethnicity, education level, marital status, employment status, income, smoking status, history of hospitalization due to complication of type 2 diabetes.

**Section B for Diabetes knowledge test questionnaire**

Section B will assess general knowledge of diabetes. Validity and reliability of study instrument the translated Malay version Diabetes knowledge test will be used for pre and post-test. For the translated questionnaire, the Cronbach's alpha was acceptable (α=0.573) with the Cronbach’s alpha excluding each item ranged from 0.517 to 0.602. Factor analysis with Varimax rotation identified 6 domains for the 18 items, with 2 to 6 items in each domain.

The score will be calculated in mean.

**Section C for Diabetes empowerment score questionnaire**

Section C will assess general knowledge of diabetes and diabetes self-care. The translated Malay version Diabetes empowerment score test will be used for pre and post-test. For the translated questionnaire, the Cronbach's alpha was acceptable (α=0.760).

A series of web-based education videos will be developed by primary care physicians, dietitian, physiotherapist, occupational therapist, and dentist in English scripts then translated into Malay, Chinese and Tamil.

Expert panellists will edit and review all the web-based educational videos and validate them using a video validation questionnaire with input from endocrinologists, primary care physicians, dietitians, physiotherapists, and occupational therapists and a few patients.

These videos discuss the following topics:

1. Definition and importance of HbA1c.
2. Complications of diabetes.
3. Instructions on self-monitoring blood glucose and the target value.
4. Diabetic medications and side effects.
5. The hypoglycaemia information
6. Diet information pertaining to healthy plates, regular meals, portion sizes, and mindful eating
7. To perform self-foot examination at home.
8. Instruction in home exercise
9. An introduction to eye examinations in diabetes and the need to monitor.
10. The importance of an annual dental check to prevent periodontitis.

Afterwards, the videos will be recorded in Malay, Chinese, Tamil and English with subtitles by the recording by the primary care team. Each video will be about 3 minutes. The technical team will edit the recording.

We will evaluate the feasibility of the videos (clarity of contents and language) with 10 participants. These participants would be type 2 diabetes patients with diverse age groups, ethnicity, and education levels. Their review and feedback will be revised in the videos, if necessary. Once the videos are finalised, they will be uploaded to the social media (e.g TikTok) private account or website for participants to access.

## Operational Definition

| **DEPENDANT VARIABLE** | **DEFINITION** |
| --- | --- |
| Diabetes knowledge | Self-reported by participant their diabetes knowledge |
| *HbA1c* | HbA1c level as measured the latest, and obtained by checking  patient’s record |
| *Blood pressure* | Blood pressure as measured most recently |
| *Fasting lipid profile* | Include HDL, LDL and TG level as measured the latest and  obtained by checking patient’s record |
| *Body Mass Index (BMI)* | BMI is calculated as the weight in kg divided by meter square  and classify according to WHO (for Asian population)  Underweight: <18.5kb/m2  Normal: 18.5 to 22.9kg/m2  Overweight: 23 to 27.49kg/m2  Obese: 27.5 to 30kg/m2  Obese II: 30 to 35kg/m2  Obese III: 35kg/m2 and above |
| *Waist circumference*  *(WC)* | Waist circumference is measure at the midpoint between the  lowest palpable rib and the top of the iliac crest. Normal waist  circumference for male is 90cm and female is 80cm. |
| **INDEPENDENT VARIABLE** | **DEFINITION** |
| **Sociodemographic profile** | |
| Age | Self-reported in years, determined from years of birth |
| Gender | As self-reported by participants |
| Ethnicity | As self-reported by participants |
| Education level | As self-reported by participants the highest education received |
| Marital status | As self-reported by participants single, married, divorce, widow. |
| Employment status | As self-reported by participants their occupation |
| Income | As self-reported by participants, will be grouped as B40, M40  and T20.  B40: RM0 – RM4849  M40: RM4850 – RM10,959 T20:  More than RM10,960 |
| Smoking status | As self-reported by participants smoker, non-smoker or exsmoker.  A smoker is considered to have been successfully quit  smoking if he has been abstinent without even a single puff of  cigarette for at least 6 months from the last cigarette (Quit date) |
| History of hospitalization  due to complication of  type 2 diabetes | As self-reported by participants their past medical history of  hospitalisation due to complication of type 2 diabetes  (Nephropathy, retinopathy, cardiovascular event and diabetes  foot ulcer or amputation) |

## Statistical Analysis Plan

#### Dependent Variables

In this study, the primary outcome of interest is diabetes knowledge and the secondary outcome is clinical outcome (HbA1c, blood pressure, fasting lipid profile, BMI, waist circumference) among intervention and control groups at baseline.

#### Independent Variables

Independent variable will be the web-based education video for the intervention group and a talk about diabetes educations for the control group

Intention to treat (ITT) method will be used for statistical analysis. Statistical analysis will be done using IBM SPSS Statistics version 27. Descriptive analysis will be conducted to check the error and distribution of data. Histogram with normal curve will be used for normality checking. Independent-t test will be used to investigate the difference of diabetes knowledge test between group. Paired t test will be used pretest and post-test after 6 months. Generalized Estimating Equation (GEE) will be used to investigate the effectiveness of diabetes knowledge and clinical outcome, adjusted with covariates. P<0.05 will be considered statistically significant.

## Risk and benefit to study participants

There is minimal risk involved in this study as the chances and magnitude of harm or discomfort anticipated in the research are not greater than those ordinarily encountered in daily life of the general population or during the performance of routine physical or psychological examinations or tests. During the study, sometimes, you may be taken more time to answer the questionnaires. There may or may not be any benefits to the participants. However, the use of web-based education videos can enhance diabetes knowledge and improve clinical outcomes (HbA1c, blood pressure, fasting lipid profile, BMI, waist circumference) among patients with type 2 diabetes. There is a potential for web-based education videos to be systematically implemented in all primary care clinics to simplify patient self-management and to reduce the risk that diabetes complications.

## Ethics of Study

This study will be conducted in compliance with ethical principles outlined in the Declaration of Helsinki and Malaysian Good Clinical Practice Guideline. Ethical approval will be obtained from MREC and NMRR prior to the start of any study related activities.

## Data storage, privacy and confidentiality

Regarding data storage and use are safeguard as follows:

All investigators and study site team involved with this study must comply with the requirements of the appropriate data protection legislation with regard to the collection, storage, processing and disclosure of personal information. The participant will not be identified through personal identifiable information but will be given a study identification number. Access to collated unidentifiable participant data will be restricted to individuals from the research team treating the participants, representatives of the sponsor(s) and representatives of regulatory authorities.

All the information we have collected in paper copies will be stored under lock and key in Universiti Putra Malaysia, while the electronic data including audio recordings and transcripts will be anonymized and can only be accessed with a secure password in laptops for this research. Only the researchers and regulatory authorities and Research & Development auditors will have access to the data. Published results will not contain any personal data that could allow identification of individual participants. Data will be stored for 5 years and will be destroyed after the storage period. When publishing or presenting the study results, the participant identity will not be revealed.

## Informed Consent/Assent Process

Participants will receive patient information sheets in English or Malay, depending on their preference. They will be given ample time (1 week) to make an informed decision if they are

indecisive. Participant can contact principal investigator if any clarification needed.

## Conflict of Interest

The investigators declare they have no conflict of interest.

## Publication Policy

Permission from the Director General of Health, Malaysia will be obtained prior to publication. No personal information will be disclosed, and subjects will not be identified when the findings of the survey are published. The results will be presented from the analysis of pool data.

## Termination of Study

Participants whom who wish to withdraw from the study can withdraw from the study anytime by informing principal investigator.

## Study Outcome

## The primary outcome of interest are diabetes knowledge and diabetes empowerment.

## The secondary outcome is clinical outcome (HbA1c, blood pressure, fasting lipid profile, BMI, waist circumference) among intervention and control groups at baseline.

|  | Baseline | Three month – follow up  (2/9/2024 to 6/9/2024) | Six month – follow up  (23/12/2024 to 31/12/2024) |
| --- | --- | --- | --- |
| Questionnaire | √ | √ | √ |
| Patients’ latest clinical profile (height, weight, BMI, waist circumference, blood pressure, latest blood test for HbA1c and fasting lipid profiles) will be taken from their latest medical records | √ |  | √ |

# References

1. IDF Diabetes Atlas 2021 | IDF Diabetes Atlas [Internet]. [cited 2024 Apr 8]. Available from: <https://diabetesatlas.org/atlas/tenth-edition/>
2. National Institutes of Health. Ministry of Health Malaysia National Health and Morbidity Survey (NHMS) 2019. Healthcare Demand; Institute for Health Systems Research: Selangor, Malaysia. 2020.
3. Lim CJ, Shahar S, Yahya HM, Teh SC, NS MN, Lim HC, MF MZ, Sallehuddin D, Mukhsan N. Level of nutritional knowledge and health awareness among diabetes mellitus patients at Cheras Health Clinic, Kuala Lumpur, Malaysia.[Tahap pengetahuan pemakanan dan kesedaran kesihatan di kalangan pesakit diabetes mellitus di Klinik Kesihatan, Cheras, Kuala Lumpur, Malaysia]. Sains Malaysiana. 2010;39.
4. Azimah M, Radzniwan R, Zuhra H, Khairani O. Have We Done Enough with Diabetic Education? A Pilot Study. Malays Fam Physician Off J Acad Fam Physicians Malays.

2010 Apr 30;5(1):24–30.

1. Tan MY. Self-care practices of adults with poorly controlled Diabetes Mellitus in Malaysia. 2009 [cited 2024 Apr 8]; Available from: <https://digital.library.adelaide.edu.au/dspace/handle/2440/57031>
2. Cheng LS, Aagaard-Hansen J, Mustapha FI, Bjerre-Christensen U. MALAYSIAN DIABETES PATIENTS’ PERCEPTIONS, ATTITUDES AND PRACTICES IN RELATION TO SELF-CARE AND ENCOUNTERS WITH PRIMARY HEALTH CARE PROVIDERS. Malays J Med Res MJMR. 2018 Jul 2;2(3):1–10.
3. Hussein Z, Taher SW, Gilcharan Singh HK, Chee Siew Swee W. Diabetes Care in Malaysia: Problems, New Models, and Solutions. Ann Glob Health. 2015 Nov 1;81(6):851– 62.
4. COVID-19 significantly impacts health services for noncommunicable diseases [Internet]. [cited 2024 Apr 8]. Available from: <https://www.who.int/news/item/01-06-2020-covid-19-significantly-impacts-health-services-for-noncommunicable-diseases>
5. Rothman R, Malone R, Bryant B, Horlen C, DeWalt D, Pignone M. The relationship between literacy and glycemic control in a diabetes disease-management program.

Diabetes Educ. 2004 Apr;30(2):263–73.

1. Kim S, Love F, Quistberg DA, Shea JA. Association of Health Literacy With Self-Management Behavior in Patients With Diabetes. Diabetes Care [Internet]. 2004 Dec 1 [cited 2024 Apr 8];27(12):2980–2. Available from: <https://dx.doi.org/10.2337/diacare.27.12.2980>
2. Kim TR, Ross JA, Smith DP. KOREA: Trends in Four National KAP Surveys, 1964-67. Stud Fam Plann [Internet]. 1969;1(43):6–11. Available from: <http://www.jstor.org/stable/1965090>
3. Ismaile S, Alhosban F, Almoajel A, Albarrak A, Househ M. Knowledge, Attitude and Practice Tools for Health Education Among Diabetic Patients. Inform Empowers Healthc Transform. 2017;250–2.
4. Hall L, Islam MS. Key considerations for understanding usability of digital health initiatives for adults with type 2 diabetes: a systematic qualitative literature review. Journal of Diabetes Science and Technology. 2023 May;17(3):833-42.
5. Rickheim PL, Weaver TW, Flader JL, Kendall DM. Assessment of group versus individual diabetes education: a randomized study. Diabetes care. 2002 Feb 1;25(2):269-74.
6. Wilson E, Wardle E v., Chandel P, Walford S. Diabetes Education: An Asian Perspective. Diabet Med. 1993;10(2):177–80.
7. Chen S, Qian D, Burström B. Two-year impact of an educational intervention in primary care on blood glucose control and diabetes knowledge among patients with type 2 diabetes mellitus: a study in rural China. Global Health Action [Internet]. 2021 Jan 1 [cited 2022 Mar 27];14(1). Available from: https://www.tandfonline.com/doi/epub/10.1080/16549716.2021.1893502?needAccess =true
8. Greenwood DA, Gee PM, Fatkin KJ, Peeples M. A Systematic Review of Reviews

Evaluating Technology-Enabled Diabetes Self-Management Education and Support. J Diabetes Sci Technol. 2017 Sep;11(5):1015–27.

1. Poduval S, Marston L, Hamilton F, Stevenson F, Murray E. Feasibility, Acceptability, and Impact of a Web-Based Structured Education Program for Type 2 Diabetes: RealWorld Study. JMIR Diabetes. 2020 Jan 6;5(1):e15744.
2. Turner L, Shamseer L, Altman DG, Weeks L, Peters J, Kober T, et al. Consolidated standards of reporting trials (CONSORT) and the completeness of reporting of randomised controlled trials (RCTs) published in medical journals. Cochrane Database of Systematic Reviews [Internet]. 2012 Nov 14 [cited 2024 Apr 8];2013(1). Available from: https://www.cochranelibrary.com/cdsr/doi/10.1002/14651858.MR000030.pub2/full
3. Lwanga SK, Lemeshow S, World Health Organization. Sample size determination in health studies: a practical manual. World Health Organization; 1991.
