# Supplementary material for: To develop online platform and determine its effectiveness in ENHANCING DIABetes knowledge among diabetes patients in primary CARE clinic (Enhancing-Diab-Care Study): Study protocol
Source: PLoS One. 2025 May 5;20(5):e0323102. doi: 10.1371/journal.pone.0323102 (PMC12052173; doi:10.1371/journal.pone.0323102)
Supplement: Attachment — Submitted filename: PONE-D-24-14861 (1)_edit (1).docx [file pone.0323102.s002.docx]

PLOS ONE

To develop online platform and determine its effectiveness in ENHANCING DIABetes knowledge among diabetes patients in primary CARE clinic (Enhancing-Diab-Care Study): Study protocol

--Manuscript Draft--

| Manuscript Number: | PONE-D-24-14861 |
| --- | --- |
| Article Type: | Study Protocol |
| Full Title: | To develop online platform and determine its effectiveness in ENHANCING DIABetes knowledge among diabetes patients in primary CARE clinic (Enhancing-Diab-Care Study): Study protocol |
| Short Title: | Enhancing-Diab-Care Study |
| Corresponding Author: | Hui Zhu Thew, Master of Family Medicine Universiti Putra Malaysia  SERDANG, SELANGOR MALAYSIA |
| Keywords: | Enhancing; diabetes; Self Care; Online platform; Primary care |
| Abstract: | Diabetes Mellitus Type 2 is tremendously growing in today's population, but it remains poorly controlled among patients. Insufficient knowledge and understanding of this disease contribute to this lack. Maintaining good diabetic self-care requires knowledge and empowerment. The COVID-19 pandemic showed a shift from non-communicable to communicable diseases, leading to patients becoming ignorant of their diabetic status. Therefore, we aim to develop an online education video platform that enables patients to enhance their knowledge about Diabetes Mellitus Type 2.  In this blocked randomised control trial, 232 type 2 diabetes mellitus patients will consent and voluntarily participate during their follow-up at public primary health care clinic. A routine diabetic follow-up in addition to a diabetic care video will be the intervention group, and diabetic follow-up without a diabetic care video will be as a control group.  Baseline questionnaires will be self-administered with their clinical profile to assess diabetes self-care knowledge and empowerment. Patients will be reminded to complete the educational video at the 3-month and 6-month follow-ups, and the aforementioned parameters will be reassessed. The data will be assessed using an independent t-test for the difference between intervention and control groups. A Paired t-test will assess the difference between the patient pre and post-intervention after 6 months. A generalised Estimating Equation will be used to investigate the effectiveness of diabetes knowledge and clinical outcome, adjusted with covariates. P<0.05 will be considered statistically significant.  Ethical principles outlined in the Declaration of Helsinki and Malaysian Good Clinical Practice Guideline will be followed. Ethical approval will be obtained from MREC and NMRR before starting any study-related activities. |
| Order of Authors: | Hui Zhu Thew, Master of Family Medicine |
|  | Ai Theng Cheong |
|  | Sazlina Shariff Ghazali |
|  | Poh Ying Lim |
|  | Aneesa Abdul Rashid |
|  | Ping Foo Wong |
|  | Yong Kui Choon |
|  | Adam Firdaus bin Dahlan |
|  | Chitra Suluraju |
|  | Nadiah Othman |
|  | Yong Wai Leng |
|  |  |

*Powered by Editorial Manager® and ProduXion Manager® from Aries Systems Corporation*

|  | Izwan Zuhrin bin Abdul Malek |
| --- | --- |
|  | Yoke Mun Chan |
|  | Chee Han Ng |
|  | Ja Shen Choy |
|  | Yan Bin Fong |
|  | Shen Horng Chong |
| Additional Information: |  |
| Question | Response |
| Financial Disclosure  Enter a financial disclosure statement that describes the sources of funding for the work included in this submission. Review the [submission guidelines](http://journals.plos.org/plosone/s/submission-guidelines#loc-financial-disclosure-statement) for detailed requirements. View published research articles from [PLOS ONE](https://journals.plos.org/plosone/) for specific examples.  This statement is required for submission and will appear in the published article if the submission is accepted. Please make sure it is accurate. | Yes |
| Funded studies  Enter a statement with the following details:   - Initials of the authors who received each award - Grant numbers awarded to each author - The full name of each funder - URL of each funder website - Did the sponsors or funders play any role in the study design, data collection and analysis, decision to publish, or preparation of the manuscript? |  |
| Did you receive funding for this work? |  |
| Please add funding details.  as follow-up to "Financial Disclosure  Enter a financial disclosure statement that describes the sources of funding for the work included in this submission. Review the [submission guidelines](http://journals.plos.org/plosone/s/submission-guidelines#loc-financial-disclosure-statement) for detailed requirements. View published research articles from [PLOS ONE](https://journals.plos.org/plosone/) for specific examples. | GP-IPM/2022/9714000 funded RM30,000 for video production and publication.  The sponsor (Inisiatif Putra Muda) was not involved in study design, data collection and analysis, decision to publish, or preparation of the manuscript. |

*Powered by Editorial Manager® and ProduXion Manager® from Aries Systems Corporation*

| This statement is required for submission and will appear in the published article if the submission is accepted. Please make sure it is accurate. |  |
| --- | --- |
| Funded studies  Enter a statement with the following details:   - Initials of the authors who received each award - Grant numbers awarded to each author - The full name of each funder - URL of each funder website - Did the sponsors or funders play any role in the study design, data collection and analysis, decision to publish, or preparation of the manuscript? |  |
| Did you receive funding for this work?" |  |
| Please select the country of your main research funder (please select carefully as in some cases this is used in fee calculation).  as follow-up to "Financial Disclosure  Enter a financial disclosure statement that describes the sources of funding for the work included in this submission. Review the [submission guidelines](http://journals.plos.org/plosone/s/submission-guidelines#loc-financial-disclosure-statement) for detailed requirements. View published research articles from [PLOS ONE](https://journals.plos.org/plosone/) for specific examples.  This statement is required for submission and will appear in the published article if the submission is accepted. Please make sure it is accurate. | MALAYSIA - MY |

*Powered by Editorial Manager® and ProduXion Manager® from Aries Systems Corporation*

| Funded studies  Enter a statement with the following details:   - Initials of the authors who received each award - Grant numbers awarded to each author - The full name of each funder - URL of each funder website - Did the sponsors or funders play any role in the study design, data collection and analysis, decision to publish, or preparation of the manuscript? |  |
| --- | --- |
| Did you receive funding for this work?" |  |
| Competing Interests  Use the instructions below to enter a competing interest statement for this submission. On behalf of all authors, disclose any [competing interests](http://journals.plos.org/plosone/s/competing-interests) that could be perceived to bias this work—acknowledging all financial support and any other relevant financial or non- financial competing interests.  This statement is required for submission and will appear in the published article if the submission is accepted. Please make sure it is accurate and that any funding sources listed in your Funding Information later in the submission form are also declared in your Financial Disclosure statement.  View published research articles from  [PLOS ONE](http://journals.plos.org/plosone/) for specific examples. | The authors have declared that no competing interests exist. |

*Powered by Editorial Manager® and ProduXion Manager® from Aries Systems Corporation*

| NO authors have competing interests  Enter: The authors have declared that no competing interests exist.  Authors with competing interests  Enter competing interest details beginning with this statement:  I have read the journal's policy and the authors of this manuscript have the following competing interests: [insert competing interests here] |  |
| --- | --- |
| * typeset |  |
| Ethics Statement  Enter an ethics statement for this submission. This statement is required if the study involved:   - Human participants - Human specimens or tissue - Vertebrate animals or cephalopods - Vertebrate embryos or tissues - Field research   Write "N/A" if the submission does not require an ethics statement.  General guidance is provided below. Consult the [submission guidelines](http://journals.plos.org/plosone/s/submission-guidelines#loc-guidelines-for-specific-study-types) for detailed instructions. Make sure that all information entered here is included in the Methods section of the manuscript. | The study was reviewed by MEDICAL RESEARCH & ETHICS COMMITTEE, MINISTRY OF HEALTH MALAYSIA. The approval number is NMRR ID-22-02453- OKN (IIR). Consent was taken writtenly. The participants information and details will be kept anonymous. |

*Powered by Editorial Manager® and ProduXion Manager® from Aries Systems Corporation*

| Format for specific study types  Human Subject Research (involving human participants and/or tissue)   - Give the name of the institutional review board or ethics committee that approved the study - Include the approval number and/or a statement indicating approval of this research - Indicate the form of consent obtained (written/oral) or the reason that consent was not obtained (e.g. the data were analyzed anonymously)   Animal Research (involving vertebrate animals, embryos or tissues)   - Provide the name of the Institutional Animal   Care and Use Committee (IACUC) or other relevant ethics board that reviewed the study protocol, and indicate whether they approved this research or granted a formal waiver of ethical approval   - Include an approval number if one was obtained - If the study involved non-human primates, add additional details about animal welfare and steps taken to ameliorate suffering - If anesthesia, euthanasia, or any kind of animal sacrifice is part of the study, include briefly which substances and/or methods were applied   Field Research  Include the following details if this study involves the collection of plant, animal, or other materials from a natural setting:   - Field permit number - Name of the institution or relevant body that granted permission |  |
| --- | --- |
|  |  |
| Data Availability  Authors are required to make the data underlying their research findings fully available, without restriction.  If the manuscript reports pilot or preliminary results, the data underlying those results must be made available at the time of publication. A Data Availability | Yes - Results are reported and the data are fully available |

*Powered by Editorial Manager® and ProduXion Manager® from Aries Systems Corporation*

| Statement describing where the data can be found is required at submission. Your answers to this question constitute the Data Availability Statement and will be published in the article, if accepted. When legal or ethical requirements prohibit public data sharing, authors must indicate how researchers may obtain access to the data. See the [PLOS Data Policy](https://journals.plos.org/plosone/s/data-availability) and [FAQ](https://journals.plos.org/plosone/s/data-availability#loc-data-policy-faqs) for detailed information. |  |
| --- | --- |
| Important: Stating ‘data available on request from the author’ is not sufficient. If your data are only available upon request, select ‘No’ for the first question and explain your situation in the text box. |  |
| For protocols without pilot or preliminary data, authors are strongly encouraged to state how they plan to share research data from their study when it is completed or published.  If your study includes pilot data, will they be made fully available without restriction? |  |
| Describe where the data may be found in full sentences. If you are copying our sample text, replace any instances of XXX with the appropriate details.   - If no pilot data are reported in the manuscript, please enter the following: No datasets were generated or analysed during the current study. All relevant data from this study will be made available upon study completion. - If the manuscript reports pilot data held in a public repository, include URLs, accession numbers or DOIs. If this information will only be available after acceptance, indicate this by ticking the box below. For example: All XXX files are available from the XXX database (accession number(s) XXX, XXX.). - If the manuscript reports pilot data contained within the manuscript and/or | No datasets were generated or analysed during the current study. All relevant data from this study will be made available upon study completion. |

*Powered by Editorial Manager® and ProduXion Manager® from Aries Systems Corporation*

| Supporting Information files, enter the following:  All relevant data are within the manuscript and its Supporting Information files.   - If neither of these applies but you are able to provide details of access elsewhere, with or without limitations, please do so. For example:   Data cannot be shared publicly because of [XXX]. Data are available from the XXX Institutional Data Access / Ethics Committee (contact via XXX) for researchers who meet the criteria for access to confidential data.  The data underlying the results presented in the study are available from (include the name of the third party and contact information or URL).   - This text is appropriate if the data are owned by a third party and authors do not have permission to share the data.   For study protocols with data management or sharing plans, authors are encouraged to briefly describe in their Data Availability Statement how data they generate will be made accessible when the study is completed. For example:   - Deidentified research data will be made   publicly available when the study is completed and published.  * typeset |  |
| --- | --- |
| Additional data availability information: |  |

*Powered by Editorial Manager® and ProduXion Manager® from Aries Systems Corporation*

Manuscript	[Click here to access/download;Manuscript;Manuscript.docx](https://www2.cloud.editorialmanager.com/pone/download.aspx?id=36155200&guid=89eb58d6-625e-4e6b-a0da-1e7ca329b132&scheme=1)[<https://www2.cloud.editorialmanager.com/pone/download.aspx?id=36155200%26guid=89eb58d6-625e-4e6b-a0da-1e7ca329b132%26scheme=1>](https://www2.cloud.editorialmanager.com/pone/download.aspx?id=36155200&guid=89eb58d6-625e-4e6b-a0da-1e7ca329b132&scheme=1)

To develop online platform and determine its effectiveness in **ENHANCING DIAB**etes knowledge among diabetes patients in primary **CARE** clinic (**Enhancing-Diab-Care** Study): Study protocol

#### Authors

Hui Zhu Thew^1^, Ai Theng Cheong^1^, Sazlina Shariff Ghazali^1^, Poh Ying Lim^2^, Aneesa Abdul Rashid^1^, Ping Foo Wong^3^, Yong Kui Choon^3^, Adam Firdaus bin Dahlan^3^, Chitra Suluraju^3^, Nadiah Othman^3^, Yong Wai Leng^3^, Izwan Zuhrin bin Abdul Malek^4^, Yoke Mun Chan^5^, Yan Bin Fong^6^, Chee Han Ng^6^, Ja Shen Choy^6^, Shen Horng Chong^6^

#### Author details

1 Department of Family Medicine, University Putra Malaysia, Serdang, Selangor, Malaysia

2 Department of Community Health, Universiti Putra Malaysia, Serdang, Selangor, Malaysia

3 Primary Health Care Clinics, Ministry of Health Malaysia

4 Department of Rehabilitation, Faculty of Medicine and Health Sciences, UPM

5 Department of Dietician, Faculty of Medicine and Health Sciences, UPM

^6^Faculty of Medicine and Health Sciences, Universiti Putra Malaysia, Serdang, Selangor, Malaysia

* Corresponding author : [jothew@upm.edu.my](mailto:jothew@upm.edu.my) (HZ)

#### Metadata Funding

This research is funded by Inisiatif Putra Muda (GP-IPM), GP-IPM/ 2022/9714000, by Universiti Putra Malaysia.

#### Competing interests

The authors declare no competing interests.

#### Data Availability

Data sharing is not applicable to this article as no datasets were generated or analysed. Data will be available after the completion of the study.

# Abstract

Diabetes Mellitus(DM)Type 2 is increasingly prevalent in today's population.DM remains poorly controlled due to insufficient knowledge and understanding of this disease. Maintaining good diabetic self-care requires knowledge and empowerment. The COVID-19 pandemic showed a shift from non-communicable to communicable diseases, leading to patients becoming ignorant of their diabetic status. Therefore, we aim to develop an online education video platform that enables patients to enhance their knowledge about Diabetes Mellitus Type 2.

In this blocked randomised control trial, 232 type 2 diabetes mellitus patients will consent and voluntarily participate during their follow-up at public primary health care clinic (one or several?refer to section on study population). A

routine diabetic follow-up in addition to a diabetic care video will be the intervention group, and diabetic follow-up without a diabetic care video will be as a control group.

Baseline questionnaires will be self-administered with their clinical profile to assess diabetes self-care knowledge and empowerment. Patients will be reminded to complete the educational video at the 3-month and 6-month follow-ups, and the aforementioned parameters will be reassessed. The data will be assessed using an independent t-test for the difference between intervention and control groups. A Paired t-test will assess the difference between the patient pre and post-intervention after 6 months. A generalised Estimating Equation will be used to investigate the effectiveness of diabetes knowledge and clinical outcome, adjusted with covariates. P<0.05 will be considered statistically significant.

Ethical principles outlined in the Declaration of Helsinki and Malaysian Good Clinical Practice Guideline will be followed. Ethical approval will be obtained from MREC and NMRR before starting any study-related activities.

# Introduction

Diabetes is a growing global health concern, with its highest prevalence found in low- and middle-income countries and accounts for 1.5 million deaths per year. In Southeast Asia, type 2 diabetes is now the most prevalent disease.

(1) According to the International Diabetes Federation (IDF), 34 million people in south-east Asia have diabetes. According to estimates, the number will rise to over 55 million by 2035. (1) In Malaysia, approximately 3.9 million people have diabetes, according to the National Health and Morbidity Survey 2019. This rate

has increased from 13.4% in 2015 to 18.3% in 2019. Alternatively, that is approximately one-fifth of the adult population in Malaysia (2). There is a high prevalence of poor glycaemic control with 7.9% mean HbA1c levels. (2) .Several studies show that Malaysians with poor glucose control engage in poor self-care practices and have a limited understanding of diabetes (3–6) .Additionally, there are also insufficient diabetes educators to deal with a large number of diabetes patients (7). Furthermore, COVID-19 has significantly impacted the entire healthcare system in recent years. Primary care and hospital practice are currently overburdened with diagnostic testing, monitoring, and management of COVID-19 care,resulting in routine maintenance for non-communicable diseases becoming compromised (8).Given these factors, there is an urgent need for practical solutions.

Studies have demonstrated that diabetes education improves self-care and glucose control among people with low health literacy. At the same time, clinicians have less time and resources to disseminate information(9,10) .The number of diabetic education classes is disappointingly low, particularly among patients with lower socioeconomic classes, those who have not yet developed diabetes complications, and during the COVID-19 pandemic when most patients are unwilling to stay in the hospital for longer.

Diabetes self-care programs include health education, a critical component for managing and treating chronic diseases(11).This principle underpins the theory that knowledge, awareness, and practices (KAP) of patients with diabetes are considered the most important factors for assessing health education outcomes. As the KAP theory outlines, human behaviour

change can be divided into three steps: acquiring knowledge, creating attitudes and beliefs, and changing behaviours, during which human health behaviours can also be transformed (12,13). The use of knowledge can assist patients in making informed decisions about their health when dealing with non- communicable diseases such as diabetes. By empowering them, patients can increase their self-efficacy and improve their self-confidence. Utilising the media in publicity campaigns, creating educational materials, and educating people with diabetes and their families should be part of this effort

(14). Diabetes education and awareness programs will benefit healthy subjects as prevention measures and help people with diabetes better control their condition. (15,16) Providing specific and practical guidance for maintaining NCDs’ critical health and community services is essential. National guidance is required to develop and use digital health solutions in NCD care, self-care, at- home care, and peer support. Yet, with the challenges of the COVID-19 pandemic facing us in the 21st century, it is vital to evaluate how medical information is delivered effectively through health promotion in the practice of new norms. The systematic reviews on using technology to facilitate diabetes self-management reported a significant reduction in glycated haemoglobin and cost-effectiveness. With internet-delivered diabetes education, many individuals have easier access to the material and can take their time with the learning process. (17–19)

Therefore, this work aims to develop and investigate the effectiveness of web- based education videos that include tailoring information on knowledge to educate type 2 diabetes patients in primary care.

# Materials and Methods

## Study design

The study design to be used in this study is a Blocked Randomized Control Trial (RCT) ratio one to one. Participants will be randomly assigned in a 1:1 ratio to either the treatment or control group to minimise biases in outcome assessment and data analysis. The data collector will remain blinded to group assignments and undergo training to adhere to standardized protocols during data collection. Additionally, random monitoring of data collection will be implemented to ensure and sustain reliability. Inclusion and exclusion criteria have been designed to control for confounding variables. The study will be conducted and evaluated by the requirement in the Consolidated Standards of Reporting Trials (CONSORT) Statement (20).

## Study population

The study population consists of type 2 diabetes patients who registered in walk-in and follow-up clinics in the primary care clinics from November 1, 2023, to May 31, 2024.

## Study phase

### Pre-development phase

An initial literature review was conducted as part of this preliminary phase

to scope out the systematic reviews and guidelines of the type 2 diabetes education online education module. In total, 12 topics were identified by the primary care physician and three patients as essential diabetes knowledge. According to our research, there are

no standard diabetes education videos available online in English, Malay, Chinese, and Tamil for healthcare practitioners dealing with type 2 diabetes.

**Fig 1. Schedule of enrolment, intervention, and assessment**. Schedule of enrolment, interventions, and assessments according to SPIRIT protocol tools

### Development phase

In this phase, a set of English-version online education videos will be developed. The scripts are based on the most recent evidence and guidelines. They are presented in a simple, short, and straightforward manner to facilitate patients' understanding of self- care for type 2 diabetes. Each video was intended to last no more than ten minutes.

### Review and finalisation phase

Following the completion of the scripts, all experts and patients were invited to participate in the review and finalisation phase. The content and validity of each video were acceptable, with a score of 0.8.

### Pilot testing phase

Each language version of the final videos will be tested with ten patients for this phase face-to-face, and their feedback will be solicited. Documentation of the sessions is being conducted to understand better how patients perceive the videos' usefulness and ease of use.

## Data collection

The researchers will collect data by selecting type 2 diabetes patients from the baseline. Only patients with type 2 diabetes who meet all inclusion criteria and do not satisfy any exclusion criteria will be recruited in our study. A research assistant will approach patients on their follow-up visit at Klinik Kesihatan Cheras Baru. As part of the consent process, the investigator will explain and provide the Patient Information Sheet to the participant and an agreement to receive calls and messages from the researcher for research purposes. They will keep their phone numbers private and only be contacted or messaged every four weeks to remind them to complete the video-watching and follow-up appointment. The patients will, however, be given one week to consider their participation if they are still deciding on the recruitment day. We will conduct a one-to- one ratio randomisation control trial with the control and intervention groups.

During the same day, all participants will be given a hard copy self-administered questionnaire by research assistance to complete, and their latest clinical profile (height, weight, BMI, waist circumference, blood pressure, latest blood test for HbA1c and fasting lipid profiles) will be taken from their medical records. The self-administered questionnaire will take about 20 mins to complete. This stage will be considered a baseline assessment for control and intervention groups. After the questionnaire, both groups will follow up in the clinic as usual. The intervention group will take place after all the participants complete the questionnaire. The participants will be told about the structure of the program. There will be research assistants the researcher has already trained to assist and teach the participants how to assess and use the web-based online education videos with a QR code. They will need to watch all the videos during the study period in their free time. The research assistant will follow up with their progress and

remind them to complete the video every four weeks via phone call. After three months of the intervention, both the intervention and control group participants will return to the clinics for follow-up. During the follow-up, participants will be given a self-administered questionnaire to answer.

Six months from the baseline, the intervention and control group participants will return to the clinics for follow-up. During the follow-up, participants will be given a self- administered questionnaire to answer, and their latest clinical profile (height, weight, BMI, waist circumference, blood pressure, latest blood test for HbA1c and fasting lipid profiles) will be taken from their medical records. As a result of this study, participants will watch a video to learn about diabetes and how to take care of themselves; no further treatment will be administered after the study is completed. Patients who wish to withdraw from the study can withdraw anytime by informing the principal investigator. The flow of the process is summarised in Fig 2.(where is this Figure 2)

## Inclusion/Exclusion criteria

The inclusion criteria of participants are as follows: 1) type 2 diabetes patients aged 18 years or older, 2) have been diagnosed and followed up for type 2 diabetes for at least six months, 3) have an HbA1c greater than 8% at the time of enrolment, 4) only one family member from each household will participate in the study, 5) able to understand Malay,

6) have internet access at home.

Exclusion criteria are as below: 1) patients who are acutely ill, 2) have psychiatric illness,

3) bedridden and rely on nursing care to perform daily activities, 3) intellectually disabled, as determined by a community-centred board.

## Study instrument

As a baseline measure, respondents will be provided with self-administered questionnaires in the Malay language upon initiation of recruitment. This questionnaire will have three sections: Section one will cover sociodemographic factors (age, gender, ethnicity, education level, marital status, employment status, income, smoking status, and history of hospitalisation for complications related to type 2 diabetes). The second section consists of a diabetes knowledge test, and the third consists of a diabetes empowerment score questionnaire.

A series of web-based educational videos will be developed by primary care physicians, dietitians, physiotherapists, occupational therapists, and dentists before assessing the general knowledge of diabetes and diabetes self-care among our respondents. After the English version, the videos will be translated into Malay, Chinese and Tamil. These videos will cover the following topics: 1) An Introduction to Enhancing Diabetes Care. 2) A guide to screening, diagnosis and HbA1c test. 3) Oral anti-diabetes medications. 4) Injection form anti-diabetes medications and injection technique. 5) Self Monitoring Blood Glucose (SMBG). 6) Hypoglycemia. 7) Dietary. 8) Self-care. 9) Exercise. 10) Understanding complications and prevention. 11) Recap and Roadmap to a better future. 12) Real-life journey: A type 2 diabetes patient shares his testimony. Before the video recording, the expert panellists will review, edit, and validate the videos using a video validation questionnaire with input from endocrinologists, primary care physicians,

pharmacists, ophthalmologists, dietitians, rehabilitation specialists, and a few patients. For the translated questionnaire, the Cronbach's alpha was acceptable (α=0.573).

## Sample size

The sample size was calculated based on two mean groups formula (21); mean±sd of knowledge differences for the intervention group and control group were 0.7±2.21 and -0.30±2.33 (17), 80% of power, 5% of risk error, 30% attrition rate, the sample size needed for each group is 116, with the total is 232.

## Statistical analysis plan

In this study, the primary outcome of interest is diabetes knowledge, and the secondary outcome is the clinical outcome (HbA1c, blood pressure, fasting lipid profile, BMI, waist circumference) among intervention and control groups at baseline. The Independent variable will be the web-based education video for the intervention group and a talk about diabetes education for the control group. The intention to treat (ITT) method will be used for statistical analysis. Statistical analysis will be done using IBM SPSS Statistics version 27. Descriptive analysis will be conducted to check the error and distribution of data. A histogram with a standard curve will be used for normality checking. Independent-t test will be used to investigate the difference of diabetes knowledge test between groups. Paired t test will be used pretest and post-test after six months. Generalized Estimating Equation (GEE) will be used to investigate the effectiveness of diabetes knowledge and clinical outcome, adjusted with covariates. P<0.05 will be considered statistically significant.

# Ethical considerations and declaration

This study adheres to ethical standards outlined in the Declaration of Helsinki and the Malaysian Good Clinical Practice Guideline. Before initiating any study-related activities, ethical approval will be obtained from MREC and NMRR. Our research study was registered under Thai Clinical Trials Registry (identification number: pilo). All investigators and the study site team must comply with data protection legislation, ensuring the confidentiality of personal information. Participants will be assigned study identification numbers to maintain anonymity.

Access to collated participant data will be restricted to individuals from the research team treating the participants, sponsor representatives, and representatives of regulatory authorities. Paper copies of collected information will be securely stored at Universiti Putra Malaysia. At the same time, electronic data, including audio recordings and transcripts, will be anonymised and accessible only with a secure password. Data will be retained for 5 years and then destroyed.

During recruitment, participants will receive patient information sheets in English or Malay based on preference, with a one-week ample period for informed decision- making. The principal investigator can be contacted if any clarifications are needed. Published results will not include identifiable personal data, and participant identities will be concealed during the presentation of study results. Participants who wish to withdraw from the study can withdraw anytime by informing the principal investigator.

# Status and timeline of study

Table 1 shows the study's timeline. At this point, no data collection has taken place.

# Discussion

Malaysia holds the highest diabetes rate in the Western Pacific region and ranks among the highest globally, contributing to an annual economic cost of approximately 600 million United States dollars. Diabetes is expected to affect 7 million Malaysian adults aged 18 and older by 2025, posing a major public health risk with a diabetes prevalence of 31.3%.

(22) The effect of improving self-care in diabetic patients on disease mortality and morbidity is significant (23).Therefore, our study is to investigate the effectiveness of web-based education videos that include tailored information on knowledge to educate type 2 diabetes patients in primary care. The findings from our study will enrich the existing literature on diabetes interventions by showing the effectiveness of web-based educational videos on knowledge of diabetes mellitus and diabetic self-care. This Randomized Controlled Trial (RCT) is an approach to improve recruitment strategies, training materials, and the implementation protocol for a larger cluster RCT in the future. The findings from this study will be shared with academics and the community of our sample population, fostering sustained improvements in diabetes outcomes and promoting health equity.

This study intends to use a new approach towards helping healthcare providers explain to patients regarding type 2 diabetes mellitus. In this modern era, people are more invested towards social media and the internet platform has become the main source of information. Therefore with the development of web-based educational videos, people

can enhance their knowledge of type 2 diabetes mellitus effectively. As a result, they will better understand their treatment options and participate in patient-centred care. Understanding the disease will allow them to better focus on their health and ease the burden of healthcare providers on managing their disease. The web-based approach will be our initial step towards making our country a well-managed diabetes centre.

Our study has certain limitations. Firstly, the sample is drawn from patients attending Klinik Kesihatan Cheras Baru, which may not represent the broader population of type 2 diabetes patients. Different socioeconomic backgrounds may reflect on our web-based approach as some may not have access to proper internet connection or smartphones. Second, the participants are possibly exposed to the Hawthorne Effect or observer bias. When patients are constantly reminded to watch a video before being tested on their knowledge, they tend to perform better than they would otherwise. In real-life clinical situations, patients aren't usually reminded as often to watch such videos. Previous study demonstrated that forgetfulness is one of the main reasons (88%) for non-compliance with treatment plans among Diabetes Mellitus patients. (24) Eliminating the forgetfulness factor by frequently calling the patient might not reflect the real effect on daily clinical practice. Next, participants may not consistently complete the videos or follow-up appointments as required, affecting the validity.

# Authors’ contributions

## Acknowledgements

The authors acknowledge the Department of Family Medicine team for their contributions to drafting this study protocol.

## Supporting Information

**S1 Fig 1. Schedule of enrolment, intervention, and assessment**. Schedule of enrolment, interventions, and assessments according to SPIRIT protocol tools

**S2 Fig 2. Flow of process**. The flow of the study was summarised as above

**S3 Table 1**. Timeline of the study. The proposed timeline for the study was described as above

**S4. Model consent form**

# References

1. IDF Diabetes Atlas 2021 | IDF Diabetes Atlas [Internet]. [cited 2024 Apr 8]. Available from: https://diabetesatlas.org/atlas/tenth-edition/
2. Institute for Public Health 2020. National Health and Morbidity Survey (NHMS) 2019: Non-communicable diseases, healthcare demand, and health literacy—Key Finding
3. Lim CJ, Suzana Shahar, Hanis Mastura Yahya, Teh SC, Nor Shazwani Mohd Nor, Lim HC, et al. Level of nutritional knowledge and health Awareness among diabetes mellitus patients at Cheras Health Clinic, Kuala Lumpur, Malaysia. 2010 [cited 2024 Apr 8]; Available from: <http://www.ukm.my/jsm/english_journals/vol39num3_2010/contentsVol39num3_2010.ht> ml
4. Azimah MN, Radzniwan R, Zuhra H, Khairani O. Have We Done Enough with Diabetic Education? A Pilot Study. Malays Fam Physician [Internet]. 2010 [cited 2024 Apr 8];5(1):24. Available from: /pmc/articles/PMC4170382/
5. Tan MY. Self-care practices of adults with poorly controlled Diabetes Mellitus in Malaysia. 2009 [cited 2024 Apr 8]; Available from: https://digital.library.adelaide.edu.au/dspace/handle/2440/57031
6. Cheng LS, Aagaard-Hansen J, Mustapha FI, Bjerre-Christensen U. MALAYSIAN DIABETES PATIENTS’ PERCEPTIONS, ATTITUDES AND PRACTICES IN RELATION TO SELF-CARE AND ENCOUNTERS WITH PRIMARY HEALTH CARE PROVIDERS.

Malaysian Journal of Medical Research (MJMR) [Internet]. 2018 Jul 2 [cited 2024 Apr 8];2(3):1–10. Available from: https://ejournal.lucp.net/index.php/mjmr/article/view/malaysiandiabetes

7. Hussein Z, Taher SW, Gilcharan Singh HK, Chee Siew Swee W. Diabetes Care in Malaysia: Problems, New Models, and Solutions. Ann Glob Health. 2015 Nov 1;81(6):851–62.
8. COVID-19 significantly impacts health services for noncommunicable diseases [Internet]. [cited 2024 Apr 8]. Available from: https://[www.who.int/news/item/01-06-2020-covid-19-](http://www.who.int/news/item/01-06-2020-covid-19-) significantly-impacts-health-services-for-noncommunicable-diseases
9. Rothman R, Malone R, Bryant B, Horlen C, Dewalt D, Pignone M. The Relationship Between Literacy and Glycemic Control in a Diabetes Disease-Management Program. Diabetes Educ [Internet]. 2004 Mar 1 [cited 2024 Apr 8];30(2):263–73. Available from:

https://journals.sagepub.com/doi/10.1177/014572170403000219?url_ver=Z39.88- 2003&rfr_id=ori%3Arid%3Acrossref.org&rfr_dat=cr_pub++0pubmed

10. Kim S, Love F, Quistberg DA, Shea JA. Association of Health Literacy With Self- Management Behavior in Patients With Diabetes. Diabetes Care [Internet]. 2004 Dec 1 [cited 2024 Apr 8];27(12):2980–2. Available from: https://dx.doi.org/10.2337/diacare.27.12.2980
11. Managing Diabetes | Self-Management Education Programs | Self-Management Education: Learn More. Feel Better. | CDC [Internet]. [cited 2024 Apr 8]. Available from: https://archive.cdc.gov/www_cdc_gov/learnmorefeelbetter/programs/diabetes.htm
12. Kim TR, Ross JA, Smith DP. KOREA: Trends in Four National KAP Surveys, 1964-67. Stud Fam Plann [Internet]. 1969;1(43):6–11. Available from: <http://www.jstor.org/stable/1965090>
13. Ismaile S, Alhosban F, Almoajel A, Albarrak A, Househ M. Knowledge, Attitude and Practice Tools for Health Education Among Diabetic Patients. Stud Health Technol Inform [Internet]. 2017 [cited 2024 Apr 8];238:250–2. Available from: https://ebooks.iospress.nl/doi/10.3233/978-1-61499-781-8-250
14. Hall L, Islam MS. Key Considerations for Understanding Usability of Digital Health Initiatives for Adults With Type 2 Diabetes: A Systematic Qualitative Literature Review. https://doi.org/101177/19322968221075322 [Internet]. 2022 Feb 7 [cited 2024 Apr

8];17(3):833–42. Available from: https://journals.sagepub.com/doi/10.1177/19322968221075322

15. Rickheim PL, Weaver TW, Flader JL, Kendall DM. Assessment of Group Versus Individual Diabetes EducationA randomized study. Diabetes Care [Internet]. 2002 Feb 1 [cited 2024 Apr 8];25(2):269–74. Available from: https://dx.doi.org/10.2337/diacare.25.2.269
16. Wilson E, Wardle E V., Chandel P, Walford S. Diabetes Education: An Asian Perspective. Diabetic Medicine [Internet]. 1993 Mar 1 [cited 2024 Apr 8];10(2):177–80. Available from: https://onlinelibrary.wiley.com/doi/full/10.1111/j.1464- 5491.1993.tb00038.x
17. Chen S, Qian D, Burström B. Two-year impact of an educational intervention in primary care on blood glucose control and diabetes knowledge among patients with type 2 diabetes mellitus: a study in rural China. Glob Health Action [Internet]. 2021 Jan 1 [cited 2024 Apr 8];14(1). Available from: https://[www.tandfonline.com/doi/abs/10.1080/16549716.2021.1893502](http://www.tandfonline.com/doi/abs/10.1080/16549716.2021.1893502)
18. Greenwood DA, Gee PM, Fatkin KJ, Peeples M. A Systematic Review of Reviews Evaluating Technology-Enabled Diabetes Self-Management Education and Support. J Diabetes Sci Technol [Internet]. 2017 Sep 1 [cited 2024 Apr 8];11(5):1015–27. Available from: https://journals.sagepub.com/doi/10.1177/1932296817713506?url_ver=Z39.88- 2003&rfr_id=ori%3Arid%3Acrossref.org&rfr_dat=cr_pub++0pubmed
19. Poduval S, Marston L, Hamilton F, Stevenson F, Murray E. Feasibility, acceptability, and impact of a web-based structured education program for type 2 diabetes: Real-world study. JMIR Diabetes [Internet]. 2020 Jan 6 [cited 2024 Apr 8];5(1):e15744. Available from: https://diabetes.jmir.org/2020/1/e15744
20. Turner L, Shamseer L, Altman DG, Weeks L, Peters J, Kober T, et al. Consolidated standards of reporting trials (CONSORT) and the completeness of reporting of randomised controlled trials (RCTs) published in medical journals. Cochrane Database of Systematic Reviews [Internet]. 2012 Nov 14 [cited 2024 Apr 8];2013(1). Available from: https://[www.cochranelibrary.com/cdsr/doi/10.1002/14651858.MR000030.pub2/full](http://www.cochranelibrary.com/cdsr/doi/10.1002/14651858.MR000030.pub2/full)
21. Lwanga SK, Lemeshow S, Organization WH. Sample size determination in health studies : a practical manual / S. K. Lwanga and S. Lemeshow [Internet]. Geneva: World Health Organization; 1991. Available from: https://iris.who.int/handle/10665/40062
22. Akhtar S, Nasir JA, Ali A, Asghar M, Majeed R, Sarwar A. Prevalence of type-2 diabetes and prediabetes in Malaysia: A systematic review and meta-analysis. PLoS One [Internet]. 2022 Jan 27;17(1):e0263139-. Available from: https://doi.org/10.1371/journal.pone.0263139
23. Selen F, Polat Ü. The effect of web based type 2 diabetes education on diabetes self management. Digit Health [Internet]. 2023 Jan 1 [cited 2024 Apr 8];9. Available from: https://journals.sagepub.com/doi/10.1177/20552076231205739
24. Rahmatullah, Qutubuddin M, Abdul Rahman R, Ghafoor E, Riaz M. Assessment of Factors Associated With Non-Compliance to Self-Management Practices in People With Type 2 Diabetes. Cureus. 2021 Oct 20;13(10):e18918. doi: 10.7759/cureus.18918. PMID: 34826318; PMCID: PMC8603089.

Figure 1	[Click here to access/download;Figure;SPIRIT-Figure.docx](https://www2.cloud.editorialmanager.com/pone/download.aspx?id=36056771&guid=195f8be8-1bb5-4e65-ae61-4042e9db26a4&scheme=1)[<https://www2.cloud.editorialmanager.com/pone/download.aspx?id=36056771%26guid=195f8be8-1bb5-4e65-ae61-4042e9db26a4%26scheme=1>](https://www2.cloud.editorialmanager.com/pone/download.aspx?id=36056771&guid=195f8be8-1bb5-4e65-ae61-4042e9db26a4&scheme=1)

Figure. RCT enrollment, intervention and assessments.

|  | **STUDY PERIOD** | | | | |
| --- | --- | --- | --- | --- | --- |
|  | **Enrolment** | **Allocation** | **Post-allocation** | | **Close-out** |
| **TIMEPOINT** | ***-t1*** | **0** | ***t1 (3 months post- allocation)*** | ***t2 (6 months post- allocation)*** | ***tx*** |
| **ENROLMENT:** |  |  |  |  |  |
| **Eligibility screen** | X |  |  |  |  |
| **Informed consent** | X |  |  |  |  |
| **Allocation** |  | X |  |  |  |
| **INTERVENTIONS:** |  |  |  |  |  |
| ***Web-based educational videos (in intervention group only)***  ***3-monthly follow***  ***up and consultation (in both intervention and control group) Video education***  ***(in both intervention and***  ***control group)*** |  |  | X | X |  |
|  |  |  | X | X |  |
|  |  |  |  |  | X |
| **ASSESSMENTS:** |  |  |  |  |  |
| ***Anthropometric and clinical profile***  ***data Diabetes knowledge and empowerment***  ***score data*** |  | X | X | X |  |
|  |  | X | X | X |  |

Figure 2	[Click here to access/download;Figure;fig.2.tiff](https://www2.cloud.editorialmanager.com/pone/download.aspx?id=36039854&guid=e5572746-32dd-4ebf-9fa1-5cc170cd0477&scheme=1)[<https://www2.cloud.editorialmanager.com/pone/download.aspx?id=36039854%26guid=e5572746-32dd-4ebf-9fa1-5cc170cd0477%26scheme=1>](https://www2.cloud.editorialmanager.com/pone/download.aspx?id=36039854&guid=e5572746-32dd-4ebf-9fa1-5cc170cd0477&scheme=1)

SPIRIT Checklist

[Click here to access/download](https://www2.cloud.editorialmanager.com/pone/download.aspx?id=36056772&guid=8581239b-2f31-49e9-ab3e-1d98862cb898&scheme=1)

[Supporting Information SPIRIT Checklist.docx](https://www2.cloud.editorialmanager.com/pone/download.aspx?id=36056772&guid=8581239b-2f31-49e9-ab3e-1d98862cb898&scheme=1)

Grant Letter (translated)

[Click here to access/download](https://www2.cloud.editorialmanager.com/pone/download.aspx?id=36166198&guid=94323c34-bafc-49d2-b879-80d1c0fdfbde&scheme=1)

[Supporting Information Translated grant letter.pdf](https://www2.cloud.editorialmanager.com/pone/download.aspx?id=36166198&guid=94323c34-bafc-49d2-b879-80d1c0fdfbde&scheme=1)

Grant Letter Original

[Click here to access/download](https://www2.cloud.editorialmanager.com/pone/download.aspx?id=36166228&guid=f2c4e6ce-d789-4614-b1c5-77e06c04df9d&scheme=1)

[Supporting Information IPM grants .pdf](https://www2.cloud.editorialmanager.com/pone/download.aspx?id=36166228&guid=f2c4e6ce-d789-4614-b1c5-77e06c04df9d&scheme=1)

Study Protocol

[Click here to access/download](https://www2.cloud.editorialmanager.com/pone/download.aspx?id=36381592&guid=3f6f3728-ffa3-4f05-80dd-e4bf3b95cfb5&scheme=1)

[Supporting Information Study Protocol Version 4.0.docx](https://www2.cloud.editorialmanager.com/pone/download.aspx?id=36381592&guid=3f6f3728-ffa3-4f05-80dd-e4bf3b95cfb5&scheme=1)
