# Supplementary material for: To develop online platform and determine its effectiveness in ENHANCING DIABetes knowledge among diabetes patients in primary CARE clinic (Enhancing-Diab-Care Study): Study protocol
Source: PLoS One. 2025 May 5;20(5):e0323102. doi: 10.1371/journal.pone.0323102 (PMC12052173; doi:10.1371/journal.pone.0323102)
Supplement: Attachment — Submitted filename: Response to Reviewers.docx [file pone.0323102.s004.docx]

| **Reviewer #1:** | |
| --- | --- |
| The authors aim to conduct a blocked randomised control trial to evaluate whether an online education video platform can enhance T2D patients’ knowledge about T2D. The intervention group will have additional routine diabetic follow-ups at 3 months and 6 months compared to the control group. The difference between the two arms and the effectiveness of diabetes knowledge will be assessed. | Thank you for your feedback. We have made amendments as per mentioned in the section: Data collection  “After three months of the intervention, both the intervention and control group participants will return to the clinics for follow-up.”  And  “Six months from the baseline, the intervention and control group participants will return to the clinics for follow-up.”  Both the control and intervention groups will not receive any additional diabetic follow-up. Patients in both the intervention and control groups will have follow-up appointments at 3 months and 6 months. The only difference is that patients in the intervention group will be provided with a diabetes care educational video and will be reminded to watch it. Routine follow-up and diabetes care provided in the clinic will be similar for both groups to avoid bias in the results. |
| 1. Some editing is needed. For example, “creating and creating educational materials…” in the introduction. | Thank you for the comment. The corresponding error has been rectified and corrected in which part, copy paste here to show them and highlight in the manuscript  “Utilising the media in publicity campaigns, creating educational materials, and educating people with diabetes and their families should be part of this effort.” |
| 2. During the review and finalization phase, all patients will be invited to participate in the review and finalization phase. Will these patients be the same or overlap with those for the later trials? If so, will this create a biased result? | Thank you for sharing your comment. These were two different groups of patients, and the patients were different and did not overlap with the trial patients. We also added in the manuscript for clarity in which part and highlight in the manuscripts under the section “review and finalization” as follow:  “ These were two different groups of patients, and the patients were different and did not overlap with the trial patients. They will review the scripts and complete a questionnaire based on the 12 video topics. ” |
| 3. Study design section claims data collector will remain blinded for group assignment. However, data collection section mentioned that the research assistant will approach patients on their follow-up visit. Please clarify these two sentences which somewhat conflicts with each other. | Thank you for pointing out the issue. Under the “study design” section, we have made the corresponding corrections under section “study design” to address this conflict  “The research assistant will undergo training to adhere to standardised protocols during data collection.” |
| 4. Will the education level or duration of T2D be considered in study design or analysis? | Thank you for your comment. Our questionnaire includes questions about both the educational level and the duration of T2DM diagnosis. The educational level is addressed in the "Latar Belakang Sosio Demografi (demography and social background)" section, question number 4, and the duration of T2DM diagnosis is covered in the "Maklumat perubatan (medical history)" section, question number 5. |
| 5. The inclusion/exclusion criteria contains “able to understand Malay”. Considering several languages will be designed, why to have this criterion? | Thank you for your comment. We include participants who are able to understand Malay because our questionnaire is administered in Malay because Malay is the official language in Malaysia. The main reason for creating the video in four languages (Malay, Chinese, Tamil, and English) is due to the multiracial background of the patients. Learning in one's own mother tongue has been proven to be more effective, as demonstrated in many studies. |
| 6. The self-administered questionnaires will be in the Malay language. But the web-based education videos will be designed in English first. Why use a different language as default? | The main video is in English, and the script is based on the most recent Clinical Practice Guidelines from Malaysia, alongside references from the American Association of Clinical Endocrinology (AACE) and the European Association for the Study of Diabetes (EASD). The information has been summarized and written in simpler terms for easier understanding. The expert panel will review the scripts and complete a questionnaire based on the 12 video topics. Each topic will be evaluated on two items: relevance and clarity, using a 3-point scale where 1 indicates 'not relevant' or 'not clear,' 2 indicates 'somewhat relevant' or 'somewhat clear,' and 3 indicates 'highly relevant' or 'highly clear.' The content and validity of each video were acceptable, with a score of 0.8. The English version was translated into Malay, Chinese, and Tamil languages, and a pilot study was conducted. A sample of 5 respondents for each language was selected. Some minor grammatical errors and difficult terminology were identified and addressed accordingly. A reliability test was conducted again for the Malay, Chinese, and Tamil languages, and the results were acceptable.  The questionnaire was administered in Malay because Malay is the official language in Malaysia. The main reason for creating the video in four languages (Malay, Chinese, Tamil, and English) is due to the multiracial background of the patients. Learning in one's own mother tongue has been proven to be more effective. Coughlin, et al (2020). |
| **Reviewer 2** | |
| Authors to clarify study site. Kindly refer to the edited attachment for language and grammar recommendations | Thank you for the comment. The corresponding grammar and language error have been rectified and corrected. |
