# Supplementary material for: To develop online platform and determine its effectiveness in ENHANCING DIABetes knowledge among diabetes patients in primary CARE clinic (Enhancing-Diab-Care Study): Study protocol
Source: PLoS One. 2025 May 5;20(5):e0323102. doi: 10.1371/journal.pone.0323102 (PMC12052173; doi:10.1371/journal.pone.0323102)
Supplement: Attachment — Submitted filename: Response_to_Reviewers_auresp_3.docx [file pone.0323102.s006.docx]

| **Reviewer #3** | |
| --- | --- |
| Abstract:  Line 3 of abstract - 'disease.. Maintaining' - double full-stop | Thank you for the comment, we had made the changes accordingly.  DM remains poorly controlled due to insufficient knowledge and understanding of this disease. |
| Line 4-'The COVID-19 pandemic showed a shift of attention from non-communicable to communicable diseases' - this statement is controversial as this statement seemed to be generalised. Is this accurate for all healthcare institutions, healthcare providers and patients? | Thank you for the comment, we had amend the sentence to make it less generalised.  The COVID-19 pandemic had caused a shift of some public attention from non-communicable to communicable diseases, leading to patients becoming ignorant of their diabetic status. |
| 2nd paragraph- Rephrase this '232 type 2 diabetes mellitus patients will consent and voluntarily participate'. Since data has not been collected, may write as the minimum sample size calculated is 232 T2DM patients. Patients will participate on a voluntary basis where informed consent will be requested prior to data collection (rephrase necessarily). | Thank you for the suggestion. We had made the relevant changes according to the suggestions.  a minimum number of 232 participants with type 2 diabetes mellitus will be needed. Patients will consent and voluntarily participate during their follow-up at public primary health care clinic (Klinik Keshihatan Cheras Baru). |
| 2nd paragraph - Suggest to rephrase 'A routine diabetic follow-up......' to - Patients undergoing diabetic follow-up will be divided into two groups where only patients within the intervention group will receive diabetic care video. | Thank you for the suggestion. We have made the changes according to the suggestion.  “Patients undergoing diabetic follow-up will be divided into two groups where only patients within the intervention group will receive diabetic care video.” |
| 3rd paragraph - 'Baseline questionnaires will be self-administered with their clinical profile to assess diabetes self-care knowledge and empowerment.' Suggest to change to 'Patients clinical profile such as (i.e give examples) were collected to assess diabetes self-care knowledge and empowerment using self-administered questionnaire.' | Thank you for the suggestion. We have made the changes according to the suggestion.  Patients' clinical profiles such as date of birth, gender, education status, Diabetes Knowledge Test (DKT) and Diabetic Empowerment Scale (DES-28) were collected to assess diabetes self-care knowledge and empowerment using self-administered questionnaires. |
| 3rd paragraph - ' reminded to complete the educational video at the 3-month and 6-month follow-ups, and the aforementioned parameters will be reassessed'- Are the patients required to revisit the videos at 3 months and 6 months? For those who have completed viewing the videos earlier, researchers will have to collect their clinical parameters only? | Thank you for the question,  All patients will be reminded to complete the educational video. If the patient had completed the video, they may choose to revise the video again to refresh their knowledge about diabetic care. |
| Study Population - Will there be any difference in terms of knowledge between walk in patients with patients followed-up (considering if they are being followed-up in another institution previously which offered them very good patient education)? relevance of duration for data collection? | Thank you for your comment. There may be differences of knowledge between walk-in patients and follow-up patients. We will conduct a one-to-one ratio randomisation control trial with the control and intervention group randomly, regardless if they are a walk-in or follow up patient. Therefore there should be no differences in knowledge level between both group |
| Development phase - State CPG which will be used? Standardise the tenses used 'The information has been summarized. In this phase, a set of English-version online education videos will be developed. They are presented in a simple, short,'. i.e will, has been, are presented. Similarly 'The English version was translated into Malay, Chinese, and Tamil languages, and a pilot study was conducted. A sample of 5 respondents for each language was selected. Some minor grammatical errors and difficult terminology were identified and addressed accordingly. A reliability test was conducted again for the Malay, Chinese, and Tamil languages, and the results were acceptable'. I think these sentences should be written as future tense. | Thank you for the comment. We have made the necessary change to specify the CPG that we used  “The main video content is in English, and the script is based on the 6th version Clinical Practice Guideline - Management of Type II Diabetes Mellitus (21)”  We had also address the the comment and change the text into future tense  “The information will be summarised and written in simpler for easier understanding. A set of English-version online education videos will be developed in this phase. They will be presented in a simple, short, and straightforward manner to facilitate patients' understanding of self-care for type 2 diabetes. Each video is intended to last no more than ten minutes.  The videos will be created from the ground up. All branding logos will be carefully designed to respect intellectual property rights, and the graphics and background music will be sourced from free, authorised channels to avoid copyright concerns. The logos will also be custom-designed to ensure originality.  Upon completion of recording and editing, each version of the videos in the four languages (English, Malay, Chinese, and Tamil) will undergo the copyright process to protect their intellectual property.” |
| Review and finalisation phase- Do you think would it be better to include certified translators as expert panels? | Thank you for your comment. Our current expert panel comprises individuals with extensive experience in both research and practical applications within the field, which has proven effective in addressing the complexities involved in our study. Your understanding of our approach is greatly appreciated. |
| Data collection -'no further treatment will be administered after the study is completed'. Can you explain what do you mean by no further treatment administered? | Thank you for your insightful comment. To clarify, no additional treatment was administered in our study. We provided the educational videos exclusively to the intervention group and fully respected the treatment decisions made by the physicians for both the control and intervention groups. |
| Study instrument- Why don't you consider translating the questionnaire into multiple languages? Are all different stages of patients going to view the same set of videos? Do you think this is suitable? | Thank you for your feedback. At present, we intend for all patients to access the same series of videos. However, we acknowledge that patients at different stages may have distinct informational needs. Our primary objective remains to ensure that all patients acquire a fundamental understanding of diabetes self-care. Tailoring video content to address specific concerns relevant to each stage could enhance understanding and relevance. We are considering this feedback seriously and may explore options for segmenting video content in future iterations of our study. |
| **Reviewer #5** | |
| Please standardize the citation style. Some citations are placed before the period, while others are after.  Ensure consistent spacing after periods throughout the document. | Thank you for the comment. The corresponding citation error has been corrected and well amended. |
| The protocol outlines several phases. Please provide detailed methods for each phase. | Thank you for your constructive feedback regarding the need for detailed methods for each phase of our study. We appreciate your emphasis on clarity and thoroughness.  Detailed Methods for Each Phase  1. Pre-Development Phase  Objective: Conduct a comprehensive literature review to identify essential diabetes education topics.  Methods:  An initial literature review was performed to scope systematic reviews and guidelines for online education modules for type 2 diabetes.  Collaborating with a primary care physician and three patients, we identified 12 essential topics for effective diabetes education.  The review indicated a lack of standardised diabetes education videos available online in English, Malay, Chinese, and Tamil.  2. Development Phase  Objective: Create a set of online education videos in English.  Methods:  The development of video scripts will be based on the most recent evidence and guidelines about diabetes care, utilising the latest Malaysian Clinical Practice Guidelines, 6th Edition, 2019, for the management of type 2 diabetes mellitus, as well as the American Diabetes Association (ADA) Diabetes Guidelines for 2024. We are committed to presenting our content clearly and straightforwardly, facilitating effective communication and understanding.  Each video is designed to last no more than ten minutes, focusing on enhancing patients' understanding of self-care for type 2 diabetes.  The following topics will be covered:   1. Introduction to Enhancing Diabetes Care 2. Guide to Screening, Diagnosis, and HbA1c Testing 3. Oral Anti-Diabetes Medications 4. Injection Form Anti-Diabetes Medications and Techniques 5. Self-Monitoring Blood Glucose (SMBG) 6. Hypoglycemia 7. Dietary Management 8. Self-Care Strategies 9. Exercise Recommendations 10. Understanding Complications and Prevention 11. Recap and Roadmap to a Better Future 12. Real-Life Journey: A Type 2 Diabetes Patient Shares Their Testimony   3. Review and Finalization Phase for English Version  Objective: Validate the content and effectiveness of the developed videos.  Methods:  Upon completion of the video scripts, we invited a panel of experts, including two endocrinologists, eight primary care physicians, two dietitians, and four patient representatives, to review the content.  A video validation questionnaire was employed to assess content validity, resulting in an acceptable score of 0.8, indicating substantial agreement on the relevance and clarity of the material.  4. Translation of Finalized English Version Video Scripts  Objective: Ensure accessibility of video content for non-English speaking audiences.  Methods:  We engaged five clinicians and four patients who are native speakers of Malay, Chinese, and Tamil and possess formal education in these languages to translate the finalised English scripts.  The translations were crafted in layman's terms to ensure the content was easily understandable for patients from diverse backgrounds.  5. Pilot Testing Phase  Objective: Test the final versions of the videos with diverse patient groups.  Methods:  Each language version of the final videos will be tested face-to-face with ten patients per language group.  Feedback regarding the videos' usefulness and ease of use will be solicited, with sessions documented for analysis. |
| Specify who verified or validated the video and indicate how many individuals were involved. | Thank you for the comment. As mentioned in the Study Phase section under the Review and Finalisation phase subsection.  “The expert panel comprises two Family Medicine Specialists, an Ophthalmologist, a Pharmacist, two Endocrinologists, a Public Health Specialist, a Rehabilitation Specialist, a Dietician, and a Chinese, a Malay, and an Indian patient. These were two different groups of patients; the patients were different and did not overlap with the trial patients. They will review the scripts and complete a questionnaire based on the 12 video topics. Each topic will be evaluated on relevance and clarity, using a 3-point scale where 1 indicates 'not relevant' or 'not clear,' 2 indicates 'somewhat relevant' or 'somewhat clear,' and 3 indicates 'highly relevant' or 'highly clear.' The content and validity of each video were acceptable, with a score of 0.8.” |
| Clarify who was involved in the translation and validation of the video into Malay, Chinese, and Indian languages. | Thank you for the comment. As mentioned in the Study Phase section under the Review and Finalisation phase subsection.  “ The English version was translated into Malay, Chinese, and Tamil by the same authors, and a pilot study was conducted. A sample of 5 respondents for each language was selected. Some minor grammatical errors and difficult terminology were identified and addressed accordingly. A reliability test was conducted again for the Malay, Chinese, and Tamil languages, and the results were acceptable.” |
| Include a reference for the scale used to assess content validity. | Thank you for your comment. We have added this in the Study phase under review and finalisation phase.  They will review the scripts and complete a questionnaire based on the 12 video topics. Each topic will be evaluated on two items: relevance and clarity, using a 3-point scale, modified from the content validity tool. (22) where 1 indicates ‘not relevant’ or ‘not clear’, 2 indicates ‘somewhat relevant’ or ‘somewhat clear’ and 3 indicates ‘highly relevant’ or ‘highly clear’. The content and validity of each video were acceptable, with a score of 0.8. The English version was translated into Malay, Chinese and Tamil languages by the same group of authors and a pilot study was conducted. A sample of 5 respondents for each language was selected. Some minor grammatical errors and difficult terminology were identified and addressed accordingly. A reliability test was conducted again for the Malay, Chinese and Tamil languages and the results were acceptable. |
| Describe the content of the diabetes education in detail. | The diabetes education video consists of the subtopic below.  1) An Introduction to Enhancing Diabetes Care.   - Describe the importance of good diabetic care.   2) A guide to screening, diagnosis and HbA1c test.   - Explains the screening test for diabetes and the introduction of HbA1c as a marker for evaluating diabetes patients   3) Oral anti-diabetes medications.   - Explains the common antidiabetic medication available and their mechanism of action   4) Injection form anti-diabetes medications and injection technique.   - Introduction of insulin injection available and the injection technique   5) Self Monitoring Blood Glucose (SMBG).   - Teach how to perform SMBG and the normal range for SMBG.   6) Hypoglycemia.   - The symptoms, prevention and management of hypoglycemia   7) Dietary.   - Dietary habits that the patient shall practise   8) Self-care.   - DFU monitoring, BP monitoring   9) Exercise.   - Introduce basic exercises that diabetic patients can do.   10) Understanding complications and prevention.   - Introduce microvascular and macrovascular complications of diabetes and the prevention of the complications.   11) Recap and Roadmap to a better future.   - A summary and motivation to the patient   12) Real-life journey: A type 2 diabetes patient shares his testimony.   - A real-life story from a T2DM patient |
| Explain how the education will be conducted and identify the individuals involved in delivering it. | Thank you for your question regarding the education delivery process and the individuals involved. The educational intervention will be conducted using a series of pre-recorded, professionally developed videos accessible through a secure link or QR code provided to participants in the intervention group. These videos are designed to be user-friendly and easily accessible on mobile devices or computers, allowing participants the flexibility to view them at their convenience. To ensure comprehension and cultural relevance, participants can select their preferred language, such as Malay, English, or Tamil.  The videos cover essential aspects of diabetes management, including understanding DM, adopting healthy lifestyle practices, monitoring blood glucose, and managing complications. The content is presented with visual aids, demonstrations, and real-life examples to enhance understanding. Participants are encouraged to watch the videos multiple times, and a designated contact person is available to address questions or provide clarification via phone or messaging.  The videos feature certified healthcare professionals, including primary care physicians, rehabilitation physician, pharmacist, dietitians, nurses, and physiotherapists, who provide expert guidance on nutrition, self-monitoring techniques, medication management, and physical activity. |
| Provide details about the questionnaires used, including their validity, language, scale, scoring, and domains.  Please help to look into the proposal and get the questionnaires and help to answer the questions | Thank you for the comment as mentioned at the Section: Study Instrument  “ This questionnaire will have three sections: Section one will cover sociodemographic factors (age, gender, ethnicity, education level, marital status, employment status, income, smoking status, and history of hospitalisation for complications related to type 2 diabetes). The second section consists of a diabetes knowledge test (DKT), which is a validated test for assessing the knowledge of a patient about diabetes, and the third consists of a diabetes empowerment score (DES-28) questionnaire which is also a validated questionnaire to assess the psychosocial self-efficacy of diabetic patients. For the translated questionnaire, Cronbach's alpha was acceptable (α=0.573). The questionnaire was administered in Malay because Malay is the official language in Malaysia. The main reason for creating the video in four languages (Malay, Chinese, Tamil, and English) is due to the multiracial background of the patients. Learning in one's own mother tongue has been proven to be more effective (21).” |
| As this is a study protocol, a discussion section is not required. However, it would be helpful to include a study flowchart and the expected results. | Thank you for the comment.  Study flowchart was included in the section “Data Collection” and a summarised form of flow chart was provided in Figure 2 for your kind perusal.  The discussion section mainly discusses the significance of the study and also the strength and limitation of this study. |
| **Reviewer #6** | |
| Elaborate on the novelty of the approach, especially how the multi-language videos address challenges in diverse populations. | Thank you for the comment. The novelty of the approach was mentioned in the discussion section which can be summarized as 3 points: 1) Adding information to the literature about educational video in control of diabetes. 2) The advantage of using internet based video as media of patient education 3)The advantage of using multilingual educational content in addressing a multiracial situation  “This study intends to use a new approach towards helping healthcare providers explain to patients regarding type 2 diabetes mellitus. In this modern era, people are more invested towards social media and the internet platform has become the main source of information. Therefore with the development of web-based educational videos, people can enhance their knowledge of type 2 diabetes mellitus effectively. As a result, they will better understand their treatment options and participate in patient-centred care. Understanding the disease will allow them to better focus on their health and ease the burden of healthcare providers on managing their disease. The web-based approach will be our initial step towards making our country a well-managed diabetes centre.  The study also intends to use a multi-language approach to address the challenge of the multicultural and multilingual population in Malaysia, especially in terms of delivery of healthcare education. Deliver educational video in their mother tongue is hopefully able to reduce the misunderstanding of communication between healthcare providers and the patients and make patient more easy to engage with the educational content.” |
| Provide more details on how the Generalized Estimating Equations (GEE) will account for covariates. | Baseline comparison between intervention and control group will be done. Significant variables will be adjusted as the covariates in Generalized Estimating Equations (GEE).  Generalized Estimating Equation (GEE) will be used to investigate the effectiveness of diabetes knowledge and clinical outcome, adjusted with covariates. Time, group, interaction of time and group variables and covariates will be included in the GEE to understand the changes of knowledge and clinical outcome over the time points between control and intervention group |
| Clarify the criteria for significant improvement in diabetes knowledge and clinical outcomes. | Thank you for the comment  The diabetes knowledge of the patient is assessed in the questionnaire administered to the patient. The section “diabetes knowledge” in the questionnaire was adopted from Diabetes Knowledge Test (DKT). A validated questionnaire to assess the knowledge of diabetes.  Clinical outcomes that measured is mentioned in the section: Data Collection  “During the follow-up, participants will be given a self-administered questionnaire to answer, and their latest clinical profile (height, weight, BMI, waist circumference, blood pressure, latest blood test for HbA1c and fasting lipid profiles) will be taken from their medical records.” |
| Compare the proposed intervention with other digital health solutions for diabetes self-management in recent studies. | Other digital health interventions for diabetes self-management include telemedicine consultations. Telemedicine serves as an effective platform for exchanging health information between patients and healthcare providers. However, it has limitations in settings with underdeveloped infrastructure and a lack of technical expertise. Bureaucratic challenges may arise due to a lack of personal contact, potentially leading to a breakdown in the relationship between healthcare providers and patients. Furthermore, a previous study conducted in the Netherlands utilized mobile health applications and demonstrated good clinical outcomes in terms of diabetes control. In this study, for patients with minimal digital skills, providing instruction and coaching on app usage is crucial as participants were required to familiarize themselves with the app and subsequently monitor and record data, including food intake and physical activity.  In our study, there will be well-trained research assistants to assist and teach the participants how to assess and use the web-based online education videos with a QR code. These research assistants will follow up their progress every 4 weeks as well. |
| Discuss how findings could influence policies or practices in resource-limited settings. | Governments and healthcare organizations could consider integrating educational videos into diabetes management programs, recognizing their cost-effectiveness and scalability. Additionally, our findings could serve as a foundation for developing standardized, evidence-based video content tailored to the cultural and linguistic needs of Malaysian populations. These videos could act as a low-cost solution to address the education gap in areas with limited healthcare worker availability. Furthermore, healthcare workers in resource-limited settings could use these videos to enhance their efforts, ensuring consistent messaging and patient education. |
| Expand on the challenges of implementing such interventions in regions with limited internet access or digital literacy. | Thank you for the comment. The limitation of limited internet access or digital literacy is mentioned in the “limitation of study” at the Section: Discussion  “Different socioeconomic backgrounds may reflect on our web-based approach as some may not have access to proper internet connection or smartphones.” |
| Consider including the potential for bias due to the Hawthorne effect or participant attrition. | Thank you for the comment. The potential for bias due to Hawthorne effect or participant attrition is mentioned in the “limitation of study” section at the Section: Discussion  “Second, the participants are possibly exposed to the Hawthorne Effect or observer bias. When patients are constantly reminded to watch a video before being tested on their knowledge, they tend to perform better than they would otherwise. In real-life clinical situations, patients aren't usually reminded as often to watch such videos. “ |
| Add a schematic overview of the intervention and assessment process for clarity. | Thank you for the comment. We included a flow diagram name Figure 2: Flow of process to display the intervention and assessment process under the section. It can be found in the attachment file under the figure or diagram section. |
